# CST1 Regulates ER-Mitochondrial Mg²⁺ Balance via MAGT1 to Promote Breast Cancer Metabolism and Growth

**DOI:** 10.7150/ijbs.136015

**Published:** 2026-09-11

**Authors:** Wenqiang Gan, Min Zheng, Li Liu, Jingwen Liao, Yu Zhu, Xiaochen Hou, Qunshan Liu, Hui Li, Jing Yao, Dewei Jiang, Ceshi Chen

**Affiliations:** 1 Key Laboratory of Animal Models and Human Disease Mechanisms of the Chinese Academy of Sciences, Kunming Institute of Zoology, Chinese Academy of Sciences, Kunming, 650201, China.; 2 Kunming College of Life Sciences, University of Chinese Academy of Sciences, Kunming, 650204, Yunnan, China.; 3 Yunnan Key Laboratory of Breast Cancer Precision Medicine, School of Biomedical Engineering, Kunming Medical University, Kunming, 650500, China.; 4 Department of Breast and Thyroid Surgery, The First Affiliated Hospital of Chongqing Medical University, Chongqing, 400016, China.; 5 Yunnan Key Laboratory of Breast Cancer Precision Medicine, Yunnan Cancer Hospital, The Third Affiliated Hospital of Kunming Medical University, Peking University Cancer Hospital, Kunming, 650118, China.; 6 Department of Breast Surgery, The First Affiliated Hospital of China Medical University, Shenyang, 110001, China.; 7 Cancer Center, Union Hospital, Tongji Medical College, Huazhong University of Science and Technology, Wuhan, 430022, China.; 8 Institute of Radiation Oncology, Union Hospital, Tongji Medical College, Huazhong University of Science and Technology, Wuhan, 430022, China.

**Keywords:** CST1, MAGT1, magnesium, metabolism, breast cancer

## Abstract

Breast cancer remains a clinical challenge and requires novel molecular targets. Here, we identify the Cystatin SN (CST1) as a critical oncoprotein upregulated in breast cancer, where its expression correlates with poor prognosis. Functionally, CST1 promotes breast cancer cell proliferation and tumor growth *in vitro* and *in vivo*, independent of its secretion. Mechanistically, CST1 physically interacts with the magnesium transporter MAGT1 on the endoplasmic reticulum (ER) membrane, and this interaction potentiates MAGT1-mediated Mg²⁺ transport, elevating mitochondrial Mg²⁺ levels and fueling mitochondrial metabolism. We developed a cell-penetrating peptide TAT-TM4_2_ which disrupts the CST1-MAGT1 interface and effectively suppresses Mg²⁺ flux, mitochondrial metabolism, and tumor growth *in vivo*. Our study unveils a non-canonical role for a secreted protein in regulating inter-organelle ion homeostasis to drive cancer metabolism and growth, and proposes a targeted peptide-based strategy for treating breast cancer.

## Introduction

The passing decades have witnessed a great number of breast cancer cases and related deaths worldwide 1-3. However, as breast cancer is highly heterogeneous, there are still huge challenges in breast cancer treatment. Based on the expression of estrogen receptors (ERα), progesterone receptors (PR) and the amplification of human epidermal growth factor receptor 2 (HER2), breast cancer can be classified into hormone receptor-positive, HER2-positive and triple-negative subtypes in clinic 4. Although hormone receptor-positive and HER2-positive breast cancers have specific targets, drug resistance is still observed 5, 6. Triple-negative breast cancers, which account for 10-20% of all cases, lack effective targets and are still dominantly treated with chemotherapy 4, 7, 8. Therefore, it is of vital importance to identifying key targets that drive breast cancer progression and designing novel and specific targeted drugs for breast cancer treatment.

Cystatin SN (CST1), a 14 kDa protein encoded by *CST1* gene, belongs to the type 2 family of the cysteine protease inhibitor superfamily and is primarily expressed in the saliva, lacrimal glands, gallbladder, semen, and prostate 9-13. CST1 has been found highly expressed in various malignant tissues, including pancreatic cancer, colorectal cancer, liver cancer, gastric cancer, and lung cancer, and is able to serve as a tumor marker 14-22. Published studies demonstrate that CST1 promotes cell cycle, proliferation, and metastasis in multiple cancers by promoting PCNA expression, activating the Wnt signaling pathway, inhibiting GSK3β phosphorylation, promoting GSH production and facilitating ROS clearance 14-22.

However. there are only limited studies on CST1 in breast tumors. Previous studies demonstrate that CST1 is upregulated in breast cancer and its high expression is corelated with worse clinical prognosis in all three breast cancer subtypes 16. It has been reported that CST1 is highest expressed in ERα+ breast cancer cell lines, promoting the activation of the ERα/PI3K/AKT/ERα loopback pathway 18. In addition, other studies suggest that CST1 is also positively regulated by transcription factor TFAP2C in ERα positive breast cancer cells and promote ferroptosis resistance by enhancing the stability of GPX4 21, 23. Recent research revealed that CST1 is upregulated in tamoxifen-resistant MCF7 and T47D cell lines, where it promotes chemotherapy resistance by inducing autophagy through RAB1B 24. However, another study indicates that CST1 is not expressed in the ERα+ breast cancer cells but in triple-negative breast cancer (TNBC) cell line MDA-MB-231 where the secreted CST1 prevents cellular senescence by inhibiting abnormal glycogen accumulation 25. Moreover, in MDA-MB-231 cells, ENO1 is reported to increase the expression of CST1 through mTOR signaling 26. These results imply that CST1 may contribute to the breast tumor progression in different breast cancer subtypes. Nonetheless, considering that the expression of CST1 in the same cell line varies in different studies, there may be fundamental flaws in previous studies on CST1. Therefore, the expression, function and mechanistic role of CST1 in breast cancer require further investigation.

Magnesium ions (Mg^2+^) are the second most abundant cation in the cell and is able to bind with different partners like ATP, ribosomes and nucleotides 27. Mg^2+^ participates in DNA stabilization, DNA repair and protein synthesis as the co-factor of a number of enzymes 27, 28. The intracellular distribution of Mg^2+^ relies on magnesium ion transporters and channels located on cellular and organelle membranes, including members of the transient receptor potential melastatin (TRPM) channel protein family, the human solute carrier (SLC) superfamily, magnesium transporter (MagT) proteins, cyclin M (CNNM) family proteins, and mitochondrial RNA splicing 2 (Mrs2) family genes 29, 30. Mg^2+^ has both promoting and inhibiting effects in tumorigenesis in various cancer types including breast cancer. Interestingly, high magnesium intake improves the prognosis of breast cancer, colorectal cancer and liver cancer 31-34. Mg^2+^ supplementation is able to induce apoptosis in colorectal cancer cells by activating the Caspase-3 pathway 35. The anti-tumor immunity of T cells relies on Mg^2+^ in lung cancer tissues as well 36. In breast cancer, Methyl Jasmonate increases intracellular Mg^2+^ concentration which leads to the increased ROS level and apoptosis 37. Besides, Mg^2+^ is also able to promote cancer proliferation. In thyroid cancer, the expressions of Mg^2+^ transporter MAGT1, NIPA1 and NIPAL1 maintain intracellular Mg^2+^ concentration and facilitate protein synthesis through the PI3K/AKT/mTOR pathway 38. Some studies suggest that higher Mg^2+^ transporters expression such as MAGT1, TRPM7, TRPM6, CNNM4 and MRS2 in various cancer types including pancreatic cancer, rectum cancer, colon cancer and breast cancer corelates with worse prognosis 28, 39.

In this study, we found that CST1 was highly expressed and functioned as an oncogene in breast cancer cell lines MDA-MB-231 and HCC1806. Mechanistically, we hypothesized that CST1 was able to regulate the Mg^2+^ balance between cytoplasm, mitochondria and ER through physically interacting with Mg^2+^ channel MAGT1 on ER member, thus promoting the mitochondria metabolism in breast cancer cells. Eventually, we developed a cell-penetrating MAGT1 derived peptide which suppressed breast cancer proliferation.

## Results

### CST1 is up-regulated in breast cancer tissues and cell lines

Cystatin superfamily, the endogenous inhibitors of C1 cysteine proteinases, contains at least 5 families, including stefin, cystatin, latexin, fetuin, and kininogen 9, 40. To investigate the expression of cystatins in breast cancer, we analyzed the TCGA database. Several cystatins were differently expressed in breast cancer tissues compared with normal breast tissues, and *CST1* was the most significant different regulated among all cystatins (Fig. 1A). The expression of *CST1* was more than 4-fold up-regulated in breast cancer tissues compared with healthy breast tissue or tumor-adjacent tissue (Fig. 1B). Using Kaplan-Meier Plotter website (https://kmplot.com/analysis/), we found that higher *CST1* expression level corelated with worse overall survival and progression-free survival in patients with systemically treatment in TCGA database (Fig. 1C-D) and GEO GSE3494, GSE20711, GSE69031 databases (Fig. S1A).

As *CST1* was up-regulated in breast tissue, we determined to find out whether it was also highly expressed in breast cancer cell lines. Using Western-blotting (WB), we were surprised that there were actually two bands near the assumed molecular weight of CST1 protein (cystatin SN), one at exactly 15 kDa (marked with *), another slightly below 15 kDa which can only be detected in three cell lines (marked with an arrow, Fig. 1E). Thought the stronger and clearer 15 kDa band seemed to be the real CST1 protein band supported by Dai 19, the RT-qPCR result which accorded with the trend of lower band supported the opposite (Fig. 1F). Moreover, knock-down and over-expression of CST1 in MDA-MB-231 and HCC1806 cell lines in which both bands exist showed that the lower band significantly changed by siRNA or plasmid transfection, while the upper bands kept unchanged or slightly changed (Fig. S1B). So, we concluded that the lower band was the real CST1 protein band, and CST1 protein was up-regulated in several breast cancer cell lines including HCC1937, HCC1806 and MDA-MB-231.

### CST1 promoted breast cancer tumorigenesis *in vitro* and *in vivo*

To confirm the function of CST1 in breast cancer, we knocked down and over-expressed CST1 in breast cancer cell lines HCC1806 and MDA-MB-231 (Fig. 2A-B). SRB and clone formation assays indicated that CST1 promoted the proliferation of breast cancer (Fig. 2C-D). However, wound healing assays suggested CST1 knock-down or over-expression cells did not affect cell migration (Fig. 2E).

We further investigated the role of CST1 in cell cycle. Knock-down of CST1 significantly arrested cell cycle in G1 phase, while over-expression of CST1 increased the cells in S phase but decreased the cells in G2/M phase without affecting G1 phase (Fig. 2F, S2A). We hypothesized that CST1 was able to accelerate DNA synthesis and confirmed it through the EdU assay (Fig. 2G, S2B). Additionally, knock- down of CST1 alone was able to induce apoptosis of MDA-MB-231 and HCC1806 cells, and over-expression of CST1 inhibited apoptosis (Fig. 2H, S2C). Furthermore, the expression level changes of proteins associated with cell cycle and apoptosis, such as CyclinD1, CyclinB1, cleaved PARP and XIAP were consisted with the flow cytometry results (Fig. S2D, S2E).

Then, we generated CST1 stable knock-down and over-expression HCC1806 cells (Fig. S2F), and injected them into the mammary fat pads of athymic nude mice. Compared with the control group, the CST1 over-expression group showed larger and heavier tumors, while the knock-down of CST1 decreased xenograft tumor growth and tumor weight (Fig. 2I-K). Taken together, these results indicated that CST1 positively regulated breast cancer tumorigenesis both *in vitro* and *in vivo*.

### CST1 promoted tumorigenesis by promoting mitochondria metabolism in a secretion independent manner

It has been reported that CST1 had a function in ROS regulation 14, 21. We decided to see whether CST1 could act as metabolism regulator of mitochondria, the primary source of cellular ROS in breast cancer. Notably, knock-down of CST1 decreased mitochondrial membrane potential, while mitochondrial membrane potential was increased when CST1 was over-expressed (Fig. 3A). As mitochondrial dysfunction may lead to the increase of intracellular ROS, we examined the ROS level in CST1 knock-down and over-expression cells. Consistently, CST1 knock-down significantly increased intracellular ROS, and CST1 over-expression showed the opposite effect (Fig. 3B). Next, we measured the mitochondrial oxidative respiration using the Seahorse XF Analyzer. The results demonstrated that knock-down of CST1 led to a decrease in overall oxygen consumption rate (OCR), as well as basal respiration, ATP production related respiration, maximal respiration and spare capacity (Fig. 3C, S3A). Consistent with the OCR result, the intracellular ATP and GSH level also had a positive correlation with CST1 level (Fig. 3D-E), which could be a consequence of the change in ROS level. The elevated ROS level can induce DNA damage, resulting in inhibited cell proliferation and ultimately leading to apoptosis. When GSH or NAC was added to the medium, the inhibition effect of CST1 knock-down could be partially rescued (Fig. S3B-C), indicating that CST1 promoted cell proliferation partially through modulating mitochondrial metabolism and ROS levels.

Next, we asked if CST1 promoted cell proliferation in a secretion dependent manner, considering CST1 is a well-known secretory protein. By analyzing cell medium, we confirmed that CST1 was able to be secreted by breast cancer cell lines MDA-MB-231 and HCC1806 (Fig. 3F). Then we generated a secretory defect mutation of CST1 by adding the ER retention signal KDEL to its C-terminus (Fig. 3G). Interestingly, the secretory defect CST1 was also able to promote breast cell proliferation (Fig. 3H), as well as decreasing intracellular ROS and increasing mitochondrial membrane potential (Fig. 3I-J). Furthermore, over-expression of CST1 in CST1 knock-down cells was able to rescue cell proliferation and ROS accumulation (Fig. S3D-F). However, extra addition of rhCST1 was unable to accelerate cell proliferation or alleviate ROS accumulation caused by CST1 knock-down (Fig. S3H-I), although rhCST1 decreased the GSK3β phosphorylation level as reported (Fig. S3G) 25. Thus, CST1 promoted breast cancer tumorigenesis in a secretion independent manner.

In order to determine the subcellular localization of intracellular CST1, we firstly isolated nucleus and cytoplasm. CST1 was localized in the cytoplasm but never in the nucleus (Fig. 3K). We further isolated organelles including mitochondrial, microsomal, or cytosolic fractions following described protocol 41 and detected CST1 localization. CST1 predominantly presented in the ER and mitochondrial fractions (Fig. 3L). As it was difficult to separate mitochondria-associated ER membranes (MAMs) from mitochondria by differential centrifugation, we co-transfected C-terminal *mEGFP*-tagged *CST1*, ER targeted *mTagBFP2* and mitochondria targeted *mRuby3* to visualize CST1 localization. Confocal laser microscopy analysis revealed that CST1 and ER-mTagBFP2 co-localized in the ER compartment (Fig. 3M). In conclusion, these results confirmed that apart from secreting, CST1 was an ER-resident protein.

### CST1 interacted with magnesium transport protein MAGT1 on ER membrane

In order to investigate the tumor-promoting mechanism of CST1, we generated C-terminal 3×Flag-tagged CST1 over-expression HCC1806 cell lines (Fig. S4A) and conducted immunoprecipitation (IP) assays by Flag M2 beads. Silver staining of the IP cell lysates revealed that CST1 was successfully pulled down (Fig. 4A). Further LC-MS showed that CST1 interacted with several ER processing factors, such as SRR3, SRR4, KDELR1 and KDELR2 (Table S1). We selected 18 proteins that might assist CST1 in tumorigenesis and performed co-IP experiments (Fig. S4B). After two rounds of co-IP, we found three proteins stably interacted with CST1, including CAV1, TMED2, and MAGT1 (Fig. S4C-D). Among three proteins, the magnesium transporter protein 1 (MAGT1) caught our attention as magnesium played a vital role in mitochondria metabolism. Exogenous and endogenesis co-IP assays confirmed that MAGT1 stably interacted with CST1 (Fig. 4B-C). Additionally, the co-IP assay by Flag M2 beads using purified C-terminal 3×Flag-tagged matured MAGT1 protein and rhCST1 supported the directly interaction between these two proteins (Fig. S4E).

Since CST1 is predominantly localized in ER, we analyzed the localization of MAGT1 by nuclear-cytoplasm separation and subcellular fractionation assays and confirmed that MAGT1 is an ER-resident protein as well (Fig. 4D-E). Additionally, by co-transfecting C-terminal *mEGFP*-tagged *CST1*, C-terminal *mScarlet-I3*-tagged *MAGT1* and ER targeted *mTagBFP2*, the co-localization of CST1 and MAGT1 in ER fractions was verified (Fig. 4F).

Next, we constructed different truncated proteins of MAGT1 to identify the domain responsible for MAGT-CST1 interaction. We divided MAGT1 into 4 fragments according to its structure (Fig. 4G). Subsequently, HEK293T cells were co-transfected with 3×HA-CST1 plasmids and MAGT1/signal peptide/TRX domain/TM1 and TM2/TM3 and TM4-3×Flag plasmids respectively, and then the co-IP was performed. The result indicated that both TRX domain and TM3/4 domain of MAGT1 interacted with CST1 (Fig. 4H). Further, we separated the two domains into more parts and co-IP analyze conformed that varies parts of TRX domain and the entire 4^th^ trans-member domain interacted with CST1 (Fig. 4I). These results indicated that MAGT1 binds with CST1 through multiple different domains.

### CST1 promotes mitochondria metabolism and breast cancer tumorigenesis through MAGT1

As MAGT1 is poorly studied in breast cancer, we analyzed its function in breast cancer firstly. The expression of *MAGT1* gene was elevated in breast cancer tissue and patients with higher *MAGT1* expression had worse prognosis (Fig. S5A-B). Moreover, similar with CST1, MAGT1 knock-down significantly inhibited proliferation of breast cancer cells lines as well as clone formation ability (Fig. S5C-F), consistent with previous published work 39. Also, MAGT1 knock-down slowed DNA synthesis confirmed by the EdU assay (Fig. S5G).

In order to demonstrate that CST1 promoted tumorigenesis in breast cancer through MAGT1, we conducted rescue experiments by knock-down MAGT1 in CST1 over-expression cells and over-expressing MAGT1 in CST1 knock-down cells (Fig. S5H). The SRB assay demonstrated that over-expression of MAGT1 in CST1 knock-down cells significantly rescued cell proliferation (Fig. 5A). Additionally, MAGT1 knock-down inhibited cell growth in CST1 over-expression cells (Fig. 5B). The clone formation ability and DNA synthesis ability were rescued by MAGT1 over-expression in CST1 knock-down breast cancer cells and MAGT1 knock-down in CST1 overexpression cell as well (Fig. 5C-F, Fig. S5J-M). The rescue effect was also confirmed *in vivo* as MAGT1 knock-down significantly inhibited the proliferation of CST1 over-expression breast xenograft tumors (Fig. 5G-H, Fig. S5I). We also observed that the mitochondrial potential membrane and ROS levels in CST1 knock-down breast cancer cells were restored when MAGT1 was over-expressed (Fig. 5I, 5K). Consistently, MAGT1 knock-down blocked CST1 over-expression induced changes of mitochondrial potential membrane and ROS levels (Fig. 5J, 5L). Taking together, we concluded that CST1 promoted breast cancer cell proliferation through MAGT1 (Fig. 5M).

### CST1 increases the magnesium transport ability of MAGT1 without altering its protein expression

Then we wondered whether CST1 regulates MAGT1 expression and activity through physically interacting. First of all, we investigated whether CST1 was able to alter MAGT1 protein level. However, knock-down of CST1 didn't significantly change MAGT1 protein level, and nor did over-expression (Fig. 6A). Considering MAGT1 was reported as a magnesium transport 42 and Mg^2+^ played an important role in mitochondria metabolism, we then determined to test the magnesium concentration under the conditions of altering CST1 expression using a cell permeant Mg^2+^ indicator, Mag-fluo-4-AM. When CST1 was knocked down in breast cancer cells, the Mag-fluo-4-AM signal enhanced (Fig. 6B), which indicated that CST1 knock-down increased Mg^2+^ levels in the whole cell. Previous studies suggested that Mg^2+^ was predominately stored in ER and it flued from ER to cytoplasm and mitochondria when cells were stimulated by metabolic product lactate through unknown Mg^2+^ transports 43. Therefore, we hypothesized that CST1 could promote the release of Mg^2+^ from ER through MAGT1. CST1 knock-down might reduce the efflux of Mg^2+^ from the ER, which leaded to the abnormal accumulation of Mg^2+^ in ER and further resulted in the upregulation of the overall intracellular but the downregulation of cytoplasm and mitochondria Mg^2+^ concentration. In order to detect Mg^2+^ concentrations in different organelles, we referred to a genetically encoded Mg^2+^ sensor, Mag-FRET (Fig. S5A) 41, 43, 44. By fusing different signal sequences, the cytoplasm located Mag-FRET protein was able to be expressed in various subcellular structures, such as nucleus, ER, and mitochondria 43, 44. To test the system, we expressed this protein in HEK293T and lysed cell by sonication. Then the cell lysis was exposed to different concentration of Mg^2+^. The excitation spectrum was measured by the microplate reader and the FRET effects were determined by fluorescence intensity ratio of Citrine and Cerulean. Similar with published results, the FRET effects enhanced with increasing Mg^2+^ concentration (Fig. S6B). Then we generated two separate ER-targeted and mitochondria-targeted Mag-FRET following previous study (Fig. S6C) 43. Notably, CST1 knock-down upregulated ER Mg^2+^ levels but downregulated cytoplasm and mitochondria Mg^2+^ levels (Fig. 6C).

Furthermore, we performed rescue assays to validate the change of intracellular Mg^2+^ concentrations caused by CST1 knock-down is through MAGT1. As expected, when MAGT1 was overexpressed in CST1 knock-down breast cancer cells, the intracellular Mg^2+^ level was significantly reduced (Fig. 6D). This result could be due to the overexpression of MAGT1 enabled the excessively accumulated Mg^2+^ within ER to be partially exported out of ER and further out of the cell. The MagFRET assay confirmed this hypothesis as MAGT1 over-expression reduced the Mg^2+^ concentration in ER but increased the Mg^2+^ concentration in mitochondria and cytoplasm (Fig. S6D). Taking together, our results indicated that CST1 was able to enhance the transport ability of ER-located MAGT1 which is responsible for Mg^2+^ release from the ER lumen to the cytoplasm and mitochondria, rather than regulating MAGT1 protein expression. The balance between the Mg^2+^ levels of ER, cytoplasm and mitochondria promoted metabolism and tumorigenesis in breast cancer cells.

### Targeting CST1-MAGT1 interaction contributed to anti-tumor therapy

Next, we tried to inhibit breast cancer proliferation by disturbing CST1-MAGT1 interaction. As there were little compounds targeting CST1 or MAGT1, we designed a cell-penetrating peptide (CCP) based on the MAGT1 fourth trans-member domain (TAT-TM4_2_) which is significant for its binding with CST1 and a negative CCP based on the second part of the TRX domain (TAT-TRX2) and synthesized CCPs by Go Top Peptide Biotech, Hangzhou, China (Fig. S7A). TAT-TM4_2_ exhibited significant cytotoxicity with IC50 values of 6.0 µM and 5.3 µM against MDA-MB-231 and HCC1806 cells respectively, while negative control TAT or TAT-TRX2 showed only modest anti-tumor effects with IC50 great than 10.0 µM (Fig. 7A). We also test the cytotoxicity of TAT-TM4_2_ in other breast cancer cell lines and normal human breast epithelial cell line MCF10A. The TAT-TM4_2_ had a broad-spectrum anti-tumor effect but exerted stronger inhibitory effects in CST1-high breast cancer cell lines MDA-MB-231, HCC1806 and HCC1937 (Fig. S7B). Over-expression of CST1 in MDA-MB-231 and HCC1806 significantly reduced the cytotoxicity effects of TAT-TM4_2_ (Fig. 7B). The knock-down of CST1 enhanced the anti-tumor effects of TAT-TM4_2_ (Fig. S7D), which might be due to superimposed effect of CST1 inhibition. To confirm TAT-TM4_2_ interacts with CST1 in breast cancer cells, we performed cellular thermal shift assay (CETSA) assays and found that 4 µM TAT-TM4_2_ protected CST1 from thermal denaturation (Fig. 7C), indicating that TAT-TM4_2_ directly bound with CST1 in cytoplasm.

However, treatment with 4 µM of TAT-TM4_2_ for 24 h had little effect in CST1 or MAGT1 protein levels (Fig. S7C), ruling out the possibility that TAT-TM4_2_ induced cell death by promoting CST1 degradation. Therefore, we hypothesized that TAT-TM4_2_ only inhibited the interaction between CST1 and MAGT1 protein. We preformed co-IP assays and showed that TAT-TM4_2_ significantly reduced the interaction between CST1 and MAGT1 (Fig. 7D). Furthermore, treatment with 4 µM TAT-TM4_2_ caused intracellular Mg^2+^ accumulation, decreased mitochondrial membrane potential, increased the ROS level, and induced breast cancer cell apoptosis, similar with CST1 knock-down (Fig. 7E-7G, S7E). Finally, we assessed the anti-tumor activity of TAT-TM4_2_* in vivo* using a xenograft model in nude mice. The mice were transplanted with HCC1806 cells into the mammary fat pad. After the xenograft tumors reached 50 mm^3^, the mice were administered TAT, TAT-TRX2 or TAT-TM4_2_ (10 mg/kg) *via* intraperitoneal injection every two days. The results indicated that TAT-TM4_2_ significantly inhibited the growth of xenograft breast tumors in *vivo* (Fig. 7H-I) without affecting the body weight of the mice (Fig. 7J). TAT-TM4_2_ treatment also showed no significant hepatotoxicity or nephrotoxicity, as indicated by serum levels of creatinine (Cr), aspartate aminotransferase (AST) and alanine aminotransferase (ALT) (Fig. 7K-M). Furthermore, immunohistochemical analysis revealed that TAT-TM4_2_ significantly reduced the expression levels of tumor proliferation marker Ki-67 index (Fig. 7N). These results suggest that TAT-TM4_2_ effectively disrupted CST1-MAGT1 interaction and inhibited the growth of breast cancer cells both *in vitro* and *in vivo* with a favorable safety profile.

## Discussion

In the present study, we identified CST1 as an oncogene in breast cancer. CST1 is up-regulated in breast cancer and promotes breast cancer cells proliferation both *in vitro* and *in vivo*, accelerates cell cycle and inhibits apoptosis but doesn't affect cell migration. Mechanistically, CST1 interacts with MAGT1 and promotes its magnesium transport ability from ER into cytoplasm. Thereby, CST1 promotes mitochondria metabolism by maintaining mitochondrial Mg^2+^ level and facilitates breast cancer tumorigenesis. Furthermore, disruption of CST1-MAGT1 interaction through a cell-penetrating peptide TAT-TM4_2_ showed anti-breast tumor activity *in vitro* and *in vivo* (Fig. 7O). Our work revealed that the CST1 protein can drive tumor progression by regulating ion transporters to influence inter-organelle ion homeostasis in a secret independent manner.

Though the roles of CST1 in different cancers have already been reported, some conclusions appear to be unreliable due to the non-specific signals of the CST1 antibody on market. The RT-qPCR results in Liu's work 18 are completely inconsistent with our result even though we used identical primer sequences. In Dai's WB results in breast cancer cells, two separate bands are observed 19. We confirmed this in this study. However, they seem to have lumped the two bands together, as they performed knockdown experiments using cells that we consider to have low CST1 expression. We agree with Oh's conclusion that CST1 was highly expressed in MDA-MB-231, but not in MCF7 cells because a home-made CST1 polyclonal antibody was used 25. Additionally, the IHC staining results in above works are also not reliable. In this work, we find that although CST1 is generally up-regulated in breast cancer cell lines, the protein expression can be only detected in cell lines including HCC1937, HCC1806 and MDA-MB-231 as the expression of CST1 is still relatively low in other cell lines. Also, the general used CST1 antibody from Proteintech has poor specificity. An anti-CST1 antibody with more specificity would assist in understanding the true expression and function of CST1 in breast tumor in the future.

CST1 has been reported to be involved in ROS metabolism in several published works. Recently, Li. showed that CST1 contributes to the stability of GPX4, a vital regulator of ferroptosis in gastric cancers 21. CST1 interacts with both GPX4 and its deubiquitinating enzyme, OTUB4, thus inhibiting the ubiquitination and degradation of GPX4. However, contrary to our results, CST1 promotes gastric cancer metastasis but not proliferation 21. Moreover, we didn't detect significant changes in GPX4 protein level in CST1 knock-down MDA-MB-231 or HCC1806 cells [data not shown]. This difference may attribute to the different subcellular localization of CST1 in different cell types. In breast cancer cells, due to the signal peptide at the N-terminal, CST1 is transferred to and localizes in ER lumen dominantly ever since being synthesized through ribosomes and is subsequently secreted 45. As a consequence, in breast cancer cells, CST1 is unlikely to interacts with GPX4 that mainly localizes in mitochondria and cytoplasm as reported 46, 47. However, in Li's research, the subcellular localization of CST1 wasn't studied except for secretion 21. In colorectal cancer, CST1 is also able to regulate GSH and ROS levels 14, but the mechanism is unknown. Recently, CST1 was also identified as glycolysis regulator in breast cancer as evidenced by seahorse assay and the changes in proteins associated with glycolysis like Glut1, HK2 and PKM2 26, which may also involve in ROS metabolism.

CST1 is a secreted protein 25. Though commonly referred to as the inhibitor of cysteine cathepsins, extracellular CST1 is able to increase the activities of CatB by competitive binding with CST3, a strong inhibitor of CatB 13, 25. Although our results confirm that the secreted CST1 inhibits the phosphorylation of GSK3β, extracellular CST1 is unable to promote the proliferation of breast cancers (Fig. S3H-I). Moreover, extracellular CST1 is reported to interact with IFNGR1 and IFNGR2, thus blocking the IFNγ signaling in macrophages and kupffer cells and establishing an immunosuppressive microenvironment in inflammation-related acute liver failure 48. The functions of secreted CST1 on breast tumor cells and other cells in the tumor microenvironment worth further investigation.

MAGT1 was originally identified as a Mg^2+^ transporter mediating Mg^2+^ uptake 42, 49. when expressed in *X. laevis* oocytes, MAGT1 specifically transports Mg^2+^ compared to other divalent cations 42. Its Mg^2+^ transporting function is also witnessed in yeast by complementation assay, or human embryonic kidney cell line HEK293t cells and human T-cells 49, 50. Furthermore, the expression of MAGT1 is increased upon low-Mg^2+^ conditions in kidney cells both *in vitro* and *in vivo* 42, 49. However, though MAGT1 has been identified for two decades, the functional characteristics of MAGT1 are poorly studied as the sequence of MAGT1 reveals no similarity to any known bacterial, yeast, or mammalian Mg^2+^ transport genes, and the structure and amino acid residue essential for Mg^2+^ transporting haven't determined yet 27, 30, 49. In addition, the loss-of-function mutations or absence of MAGT1 lead to impaired N-glycosylation of immunity related and platelet proteins, such as NKG2D, CD28 and SPARC, leading to primary immunodeficiency and platelet dysfunction as found in X-linked immunodeficiency with magnesium defect (XMEN) disease 51-54. Indeed, MAGT1 is confirmed to be a subunit of OST-B complex and is assumed to facilitate N-glycosylation by formatting mixed disulfides between its TRX domain and the glycoprotein substrates 55, 56. Although Supplementation of Mg^2+^ partly normalized the functions of platelets, NK cells and CD8+ T cells in XMEN cell models 36, 57, Mg^2+^ supplementation has not proven successful in clinic XMEN patients 58, 59. Thus, some assume that MAGT1 may not dominantly function as a Mg^2+^ transporters and it may contribute to Mg^2+^ homeostasis by modulating Mg^2+^ channels or transporters via N-glycosylation 30, 59. Although in present study, we find that CST1 is able to regulate the Mg^2+^ transporting ability of MAGT1, we cannot rule out the possibility that the changes in Mg^2+^ flux are a consequence of N-glycosylation of other Mg^2+^ channels or transporters by CST1 through MAGT1.

As the second most abundant cation in the cell, Mg^2+^ participates in almost all cellular processes such as DNA stabilization, DNA repair and protein synthesis, and is involved in multiple cell life activities such as cell proliferation, differentiation and survival 27, 28, 59. The synthesis of ATP in mitochondria requires the assistance of magnesium ions, and ATP also needs to bind with magnesium ions to perform its energy-supplying function 60, 61. Therefore, magnesium ion homeostasis is closely related to cellular metabolism. Recently, lactate is reported to trigger ER Mg^2+^ release and promotes mitochondrial Mg^2+^ uptake leading to the dysfunction of mitochondria 43. The excessive efflux of Mg^2+^ in mitochondria by CNNM4 also leads to mitochondrial dysfunction and abundant ROS production 62. Additionally, TUSC3, which belongs to the same family as MAGT1, was reported to interact with ERMA and form a complex that responsible for Mg^2+^ from cytoplasm to ER. TUSC3 knockout leads to Mg²⁺ depletion in ER, activating the PERK-eIF2α pathway and leading to ER stress 63. Furthermore, recent reports have also shown that LPS-induced magnesium efflux can cause mitochondrial damage, thereby promoting pyroptosis 64. In addition, cellular magnesium decrease is able to NF-κB-dependent cell death 65. Thus, Mg^2+^ imbalance may cause severe cellular metabolism disorder. Similarly, Methyl Jasmonate is able to upregulate intracellular Mg^2+^ level, induce ROS production and elevate ER stress, thereby promoting apoptosis in breast cancer cell line MCF7 37. In this study, inhibiting Mg^2+^ uptake in mitochondria by TAT-TM4_2_ or CST1 knock-down contributes to mitochondrial dysfunction and subsequent cell proliferation inhibition and cell death. Therefore, manipulating the intracellular distribution of metal ions such as Fe^2+^ (ferroptosis), Cu^2+^ (cuproptosis) and Na^+^ (necrosis by sodium overload, NECSO) may hold significant value for tumor treatment 66-68. Additionally, Mg^2+^ and MAGT1 have a great influence in immune function 36, 53, 69, and CST1 plays a role in immune 40, 48.

Finally, based on the structure responsible for MAGT1-CST1 interaction, we designed and synthesized a cell-penetrating peptide, TAT-TM4_2_ and succeeded in inhibiting breast cancer cell proliferation. Due to the ability to carry proteins or other biomolecules into cells, cell-penetrating peptides can serve as drug delivery vehicles 70. By mimicking interaction regions, peptides can be an effective approach to target protein-protein interactions 71. Cell-penetrating peptides linked peptides have shown great clinical potential in various diseases including tumors 72. Therefore, our study on cell-penetrating peptides provides a theoretical foundation for targeting protein-protein interactions to disrupt intracellular ion balance and for the clinical development of other anti-cancer cell-penetrating peptides. In summary, our research also offers a theoretical basis for the clinical translation and application of targeting CST1.

## Materials and Methods

### Cell lines and cell culture

All cell lines used in this study were sourced from American Type Culture Collection (ATCC, Manassas, VA, USA) and validated via short tandem repeat (STR) analysis. The breast cancer cell lines HCC1806, HCC1937, BT549 and T47D cells was cultured in RPMI1640 (Gibco, USA) with 10% fetal bovine serum (FBS, ExCell Bio, China), MCF7, MDA-MB-468, HS578T and human embryonic kidney cell line HEK293t cells was cultured in DMEM (Gibco, USA) with 10% FBS, MDA-MB-231 cells was cultured in DMEM/F12 (Gibco, USA) with 10% FBS, SUM149PT cells was cultured in F12 (Gibco, USA) with 10% FBS, 0.05 mg/mL insulin (PEProtech, USA), 1 μg/mL hydrocortisone (Sigma, USA) and 10 mM HEPES (Hyclone, USA), human breast epithelial cell lines MCF10A, 184A1 and 184B5 cells cultured in DMEM/F12 with 5% horse serum (HBS, Gibco, USA), 20 ng/mL epidermal growth factor (EGF, PEProtech, USA), 100 ng/mL cholera toxin (Sigma, USA), 8 μg/mL insulin and 500 ng/mL hydrocortisone. All cells were maintained at 37 °C in an incubator containing 5% CO_2_/95% air.

### Small interfering RNAs (siRNAs), plasmid, transfection and lentivirus construction

The siRNAs, synthesized by Qingke Bio, were transfected at a final concentration of 10 nmol/L. The target sequences for CST1 were: siCST1-2#: CAGAAGGTCCCTGGTGAAA, siCST1-4#: GGTACTAAGAGCCAGGCAA, for MAGT1 were: siMAGT1-1#: GCUAUGACAUCUGGUCAAA, siMAGT1-3#: GAGAUGGUGUUAUCUGAAA. All over-expression plasmids used in this article were generated by cloning corresponding cDNA (*CST1* transcript: NM_001898.3, *MAGT1* transcript: NM_001367916.1) into expression vector pLVX at its multi-clone site. The knock-down vectors were generated according to the pLKO.1-TRC manufacturer's instruction by addgene. The target sequences for shCST1-7# and shCST1-8# were the same with siCST1-2# and siCST1-4# respectively. Plasmid or siRNA transfection for breast cancer cells was performed using Opti-MEM (Gibco, USA) and Lipofectamine 2000 (Invitrogen) according to the manufacturer's instructions. Lentiviruses were packaged in HEK293T cells using PEI (polyethylenimine, Plybiosciences). The mass ratio for expression/knock-down plasmid, packaging plasmids psPAX2 and pMD2.G was 4:3:1. The lentivirus was harvested 48 h and 72 h post-transfection. To generate stable expression or knock-down cells, after 48 h of infection, the cells were screened with 1 μg/mL puromycin for 48 h. The screened cells were subsequently used in various assays within 8 days.

### Western blotting (WB)

After treatment, cells were washed with phosphate buffered saline (PBS) and then the cells were lysed in radioimmunoprecipitation assay (RIPA) buffer supplemented with protease and phosphatase inhibitor cocktail in 4 ℃ for 1 h. After being centrifuged at 12000 rpm in 4 ℃ for 10 min, the protein concentrations were quantified with the Pierce™ BCA Protein Assay Kit (ThermoFisher scientific, USA). The lysis was mixed with 4×sodium dodecyl sulfate (SDS) loading buffer and sit in room temperature for 15 min to avoid member proteins polymerization in high temperature 73. 30 μg of total proteins were separated via SDS-PAGE and transferred onto a PVDF membrane. The membrane was blocked in 5% non-fat milk for 1 h and incubated with the primary antibody at 4 °C overnight, followed by incubated with horseradish peroxidase-conjugated secondary antibody at room temperature for 1 h. After enhanced chemiluminescence (ECL) using an ECL reagent (UE, S6009), signals were detected through ImageQuant LAS4000 (GE, Germany).

The antibodies against CST1 (16025-1-AP, used at 1:1000) and MAGT1 (17430-1-AP, used at 1:1000) was purchased from Proteintech (Wuhan, China). Antibodies against AIF (4642, used at 1:1000), Calnexin (2433, used at 1:1000), Cyclin B1 (sc-245, used at 1:1000), Cyclin D1 (55506, used at 1:1000), Phospho-p38 MAPK (Thr180/Tyr182) (4511s, used at 1:1000), LaminB1 (13435, used at1:1000), PARP (9542, used at 1:1000), XIAP (2042, used at 1:1000) and MCL-1 (4572, used at 1:1000) was purchased from Cell Signaling Technology (Boston, USA); Tubulin antibody (T5168, used at 1:1000) and Vinculin antibody (V9131, used at 1:1000) was purchased from Sigma-Aldrich (St. Louis, MO, USA); FLAG antibody (T201126-3A6, used at 1:2000) was purchased from Zenbio (Chengdu, China); HA antibody (T008, used at 1:2000) was purchased from Affinity (Changzhou, China).

### Quantitative real-time PCR (RT-qPCR)

Total RNA was isolated from breast cancer cells using a Trizol (Invitrogen, USA) standard protocol. The RNA was examined and quantified using Nano Drop 2000 Spectrophotometer (ThermoFisher Scientific). 1 μg RNA was reverse transcribed using Hisscript II QRT Supermix (Vazyme, China). Real-time quantitative PCR reactions were then performed on an Archimed X4 (ROCGENE, China) using SYBR Green Master Mix (Vazyme). Relative gene expression levels were analyzed using comparative Ct methods where Ct was the cycle threshold number normalized to GAPDH or ACTB.

The primers for CST1 (F1: ACAAGGCCACCAAAGATGAC and R1: GGGCTGGGACTTGGTACATA for CST1 knock-down; F2: ACAAGGCCACCAAAGATGAC and R2: GGGCTGGGACTTGGTACATA for CST1 over-expression), MAGT1 (F: CTCAGCCTCTGCCCAAAGAA and R: CACAAGGCGACGGAACTTGT), GAPDH (F: CAATGACCCCTTCTTGACC and R: TTGATTTTGGAGGGATCTCG), ACTB (F: GTCTTCCCCTCCATCGTG and R: AGGGTGAGGATGCCTCTCTT) were synthesized by Tsingke Biotechnology (China).

### Protein purification

The DNA fragments that encode prokaryotes signal peptide pelB fused MAGT1 mature protein (30-335) with N-terminal 3×Flag and 6×His-tag were cloned into the Escherichia coli expression vector pEASY-Blunt (TransGen, China). The vector was transfected into E. coli DL-Rosetta (DE3) competent cells (Tsingke Biotechnology, China). The bacteria were cultured in LB broth (10 g/L NaCl, 10 g/L tryptone and 5 g/L yeast extract). When the OD600 reached about 0.6, 0.1 mM IPTG was added to the culture, followed by incubation at 16 °C, 160rpm for about 18 h. The culture was pelleted by centrifugation and resuspended in His buffer (50 mM Tris-HCl pH 8.0, 500 mM NaCl, 10%vol glycerol, 1%vol triton X-100) and lysed by sonication using Sonics Vibra-CellTM (Sonics, USA). The supernatant of the lysis was incubated with anti-Flag M2 affinity gel (sigma) for 2 h at 4 °C on a mixer as the MAGT1 recombined protein seemed to have a low affinity for the Ni-NTA column. The agarose beads were washed with 50 mL wash buffer (PBS with 1%vol triton X-100) for 2 h at 4 °C on a mixer twice. Then MAGT1 recombined tagged proteins were eluted with 5 mL elution buffer (0.1 M glycine, pH 3.0), and the eluate was immediately neutralized with 3 mL Tris-HCl (pH 8.0). The protein was detected by WB with anti-FLAG antibodies as the MAGT1 recombined protein was hard to be stained by Coomassie Blue staining.

### Co-Immunoprecipitation (Co-IP)

The HEK293t cells was used in exogenous co-IP assays. Two days post plasmids transfection, the HEK293t cells were lysed with IP buffer (150 mM NaCl, 2 mM EDTA, pH 8.0, 5 mM Tris-HCl, pH 7.4, 0.2%vol NP-40, and protease inhibitor cocktail). The lysis was centrifuged at 12000 rpm in 4 °C for 10 min to remove un-lysed cells. The supernatant was precipitated with anti-Flag or anti-HA magnetic beads (MedChemExpress, MCE, USA) for 2 h at 4 °C on a mixer. Then the beads with precipitated protein was washed for five times with IP buffer. For each washing, the beads were mixed for 10 min at 4 °C on the mixer. The beads were denatured with 4×SDS loading buffer and subjected to western blotting analysis.

For endogenous co-IP, MDA-MB-231 and HCC1806 cell lysis was precipitated with anti-CST1 antibody overnight at 4 °C and mixed with protein A/G magnetic beads (MCE) for another 2 h at 4 °C followed by five times washing. The rest steps were same as in exogenous co-IP.

For purified proteins, the MAGT1-3×Flag and rhCST1 proteins were mixed and incubated overnight at 4 °C and mixed with anti-Flag M2 affinity gel (sigma) for another 2 h at 4 °C followed by five times washing. The rest steps were same as in exogenous co-IP.

For co-IP-mass spectrum, the 3×Flag tagged CST1 over-expression MDA-MB-231 cell lysis was mixed with anti-Flag M2 affinity gel (sigma) for 2 h at 4 °C on a mixer. The precipitated proteins were washed down by flag peptide (sigma) overnight. The mixtures were subjected to NuPAGE® Novex 4-12% Bis-Tris Gel (ThermoFisher scientific) followed by silver stain (ThermoFisher scientific). The differential protein bands were cut and collected to 1.5 ml microcentrifuge tubes depend on protein observed molecular. The LC-MS/MS analysis was performed by Shanghai Institute of Materia Medica, Chinese Academy of Sciences.

### Cell viability assays

The cell viability was measured by two methods in this article. A specific number of cells were seeded into a 96-well plate. For cell growth assays, the cells were fixed by 100 µL of 10% trichloroacetic acid (TCA) day by day or at the third day after adhesion and left it at 37 °C overnight. The TCA was washed off with deionized water (dH2O) and allowed the wells to dry. Then the wells were stained with 4% Sulforhodamine B (SRB) solution for 30 min, and washed with 1% acetic acid and dried. Finally, 100 µL of 1mM Tris-base solution was added to dissolve the SRB dye and the absorbance at 530 nm was measured using a microplate reader (Infinite M200 Pro, Tecan).

For drug sensitivity assays, the CCK8 assays were performed according to the manufacturer's manual (Beyotime Biotechnology, Shanghai, China). After adhesion, the cells were treated with compounds for 2 days. Then the supernatant was removed and 100 µL 10%vol CCK8 in cell medium was added to each well. The plates were incubated for another 2 h to 4 h at 37 °C depend on cell types. The absorbance at 450 nm was determined using a microplate reader.

### Colon formation assay

2000 cells were seeded into each well of a 6-well plate. The cells were cultured for approximately 2 weeks and the medium was frequently changed until the cell colonies were obvious. Then the cells were fixed using 4% paraformaldehyde and stained with 0.1% crystal violet solution. The individual colonies containing more than 50 cells were counted.

### EdU DNA synthesis analysis

The DNA synthesis of breast cancer cells was measured using the 5-ethynyl-20-deoxyuridine (EdU) assay kit (UElandy, China). Over-expression or knock-down cells were seeded into the 6-well plates with coverslips. After adhesion, the cells were labeled with 10 μM EdU buffer at 37 ℃ for 6 h, and then fixed with 4% paraformaldehyde (Biosharp, China) in 4 °C overnight. The fixed cells were incubated with 2 mg/mL glycine for 5 min, and then washed with PBS containing 3% BSA for 2 times. The cells were permeabilized with 0.5% Triton X-100 for 20 min and blocked with 3% bovine serum albumin (BSA). The Click-iT reaction cocktail was added and the cells were incubated in the dark for 30 min. The nucleuses were stained with Hoechst 33342 solution for 15 min. The slips were sealed with an anti-fluorescence quencher. After image acquisition by ZEISS Axio imager A2 (Zeiss, Germany) using 20x objective, Image J software was used to calculate the proportion of EdU-positive cells.

### Wound healing assay

The MDA-MB-231 and HCC1806 cells were seeded in 6-well plates at 1.2 × 10^8^ cells per well and culture for 24 hours to reach full confluence. Then we created a scratch wound in each well using a sterile 200 µL pipette tip. The wells were washed three times with PBS to remove dislodged cells. And serum-free medium was added to each well. Images of the wounds at 0 ,12 and 24 hours were acquired using Nikon Eclipse Ti-S (Nikon, Japan) using 20x objective. Image J software was used to quantify cell migration by measuring the wound area.

### Cell cycle analysis

Cells were seeded into 6-well plates and treated with serum free medium for 24 h to synchronize the cell cycle. The serum starved cells were cultured with ordinary medium for 2 more days and then digested by 0.25% trypsin. The cells were wash with PBS and fixed with 75% ethanol overnight at 4 ℃. The cells were subsequently treated with 100 μg/mL RNase solution and stained with propidium iodide (PI) for 30 min in cold 0.6% NP-40 solution. DNA content was analyzed using a BD LSRFortessa Flow Cytometer (BD Biosciences, USA) and analyzed using FlowJo software.

### Apoptosis analysis

Cells were seeded into 6-well plates. After treatment, both floating and adherent cells were collected. The cells were washed with PBS, and staining with FITC/Annexin V and PI following the manufacturer's instructions of Annexin Detection kit (1133534, BD Pharmingen, USA) on ice. Apoptosis rates were measured using the Accuri C6 Flow Cytometer (BD Biosciences) and analyzed using FlowJo software. The FITC/Annexin V and PI signals were detected in in FL-1 and FL-2 channels respectively.

### ROS detection

Intracellular ROS levels were measured using the Reactive Oxygen Species Assay Kit (S0033, Beyotime). In brief, cells in 6-well plates were incubated in serum-free medium containing the fluorescent probe DCFH-DA (1:1000 dilution) for 1 h. Then the cells were digested with 0.25% trypsin, washed and resuspended in PBS. The fluorescence intensity was detected by the Accuri C6 Flow Cytometer in FL-1 channel and analyzed using FlowJo software.

### Determination of the mitochondrial membrane potential

After treatment, cells were incubated with 0.5 μg/ml of JC-1 (MCE) for 15 min at 37 °C in an incubator (5%CO_2_). Then the cells were washed with PBS and collected. The fluorescence intensity was detected by the Accuri C6 Flow Cytometer in FL-1 and FL-2 channels and analyzed using FlowJo software.

### Determination of the intracellular GSH level

Intracellular GSH levels were measured using the GSH and GSSG Assay Kit (S0053, Beyotime). In brief, cells resuspended in protein removal reagent M solution in a ratio of 30 µL M solution per 10 µg cell were subjected to freeze-thaw cycles for lysis, followed by centrifugation at 10,000 g for 10 min in 4 °C to obtain the supernatant. The reaction mixture was prepared according to the protocol. After reaction at room temperature for 5 min, absorbance at 412 nm was measured by BioTek Synergy H1 (BioTek, USA), and the GSH content in 1 g cell was calculated based on the standard curve.

### Determination of the intracellular ATP level

Intracellular ATP levels were measured using the ATP Assay Kit (S0026, Beyotime). In brief, cells were lysed by ATP cell lysis buffer at 4 °C for about 30 min. The lysis was collected and centrifugated at 12,000 g for 10 min in 4 ℃ to obtain the supernatant. The ATP detection working mixture was prepared by mixing ATP detection reagent and ATP detection reagent diluent in a 1:9 ratio. Then 20 µL cell lysis supernatant and 100 µL ATP detection working mixture were added to a well of 96-well black microplate. After reaction at room temperature for 5 s, the luminescence intensity was measured by BioTek Synergy H1 (BioTek, USA), and the ATP content in the samples was calculated based on a standard curve. To eliminate errors caused by protein concentration, the protein concentration in the supernatant was measured using the BCA method, and the ATP concentration was converted to nmol/mg protein.

### Cell mito stress test

The mitochondrial metabolism was testes by O_2_ consumption rate (OCR). The OCR assay was conducted using the Seahorse XFe24 Analyzer (Agilent Technologies). HCC1806 and MDA-MB-231 cells were seeded into the Seahorse XF24 Cell Culture Microplate at 3 × 10^4^ cells per well the day before the assay with the appropriate cell culture growth medium. The sensor cartridge of the Seahorse XFe24 Extracellular Flux Assay Kit was hydrated with the XF Calibrant (pH 7.4) at 37 °C in a CO2 free incubator overnight at the same time. The next day, the Seahorse XF24 Cell Culture Microplate was washed and changed with fresh-mixed and pre-warmed Seahorse FX DMEM medium (103575-100, Agilent Technologies) with 10 mM glucose (103577-100, Agilent Technologies), 1 mM pyruvate (103578-100, Agilent Technologies) and 2 mM glutamine (103579-100, Agilent Technologies). The cell culture was then incubated at 37 °C in a CO_2_ free incubator for 1 h. The oligomycin, carbonyl cyanide 4-trifluoromethoxyphenylhydrazone (FCCP) and a mix of rotenone and antimycin (Rot/AA) in the Seahorse XF Cell Mito Stress Test Kit (Agilent Technologies, 103015-100) were solute by the Seahorse FX DMEM medium and loaded into A, B and C ports of a sensor cartridge respectively. The final concentration of oligomycin, FCCP and Rot/AA was 2.0 μM, 1.0 μM, and 0.5 μM, respectively. The plates were analyzed by the Seahorse machine according to the manufacturer's instructions. Data were normalized against cell protein level.

### Measurement of the intracellular Mg^2+^ concentration

The breast cancer cells MDA-MB-231 and HCC1806 were cultured in 6-well plates and transfected with siRNAs or treated with TATs for 24 h. Then the cells were loaded with 1 μg/mL Mag-Fluo-4, AM (Ex/Em 495/515, ThermoFisher scientific) in serum-free medium at 37 °C in an incubator (5%CO_2_). The cells were washed with PBS and collected. The fluorescence intensity was detected by the Accuri C6 Flow Cytometer in FL-1 channels and analyzed using FlowJo software.

### Determination of the Mg^2+^ relative concentration by FRET system

The sequence of basic MagFRET was reported by Soares et al 44. To generate MagFRET stable expression cells, we cloned the basic MagFRET into pLVX vector and conduced synonymous mutations on the Citrine sequence of the MagFRET to avoid homologous recombination when packing into the lentivirus. The mitochondrial and ER targeted MagFRET was generated and reported by Daw et al 43. Generally, for ER-MaagFRET, the ER signal sequence MLLPVLLLGLLGAAAD was added to the N-terminal and ER retention sequences (KDEL) were added to the C-terminal of MagFRET. For Mio-MagFRET the mitochondrial targeting sequence (MRKMLAAVSRVLSGASQKPASRVLVASRNFANMLHFCSRRYRGPGIHR) was added to the N-terminal of MagFRET. Cells were infected with MagFRET lentivirus and screened with 1 μg/mL puromycin for 48 h. After transfected with siRNAs, the stable expressing cells were digested and resuspended in PBS. The cell suspension was added to the 96-well black microplate at 100 µL per well. The intensity of fluorescence signal from 450 nm to 600 nm excited by 420 nm excitation light was measured by BioTek Synergy H1 (BioTek, USA).

### Extraction of secretory protein

The MDA-MB-231 and HCC1806 cells were cultured in 6-well plates and treated with serum free medium for 24 h to eliminate effect from FBS. The medium was collected to a 1.5 mL microcentrifuge tubes and centrifuged at 15000g for 10 minutes at 4 °C to remove suspension cells. Then, 500 μL supernatant was transferred to a new tube and same volume of methanol was added. The mixture was added with 200 μL chloroform and mixed by vigorous shake. After centrifuging at 15000g for 10 minutes at 4 °C, a three-layer separation was observed with the denatured protein at the middle solid layer. The upper aqueous layer was then removed and cold ethanol was added to dissolve the bottom organic layer and precipitated the protein. The pellets were washed with cold ethanol and centrifuged at 15000g for another 10 min at 4 °C. After the pellets were air-dried, 8 M urea was used to dissolve the protein. The protein solution was mixed with 4×SDS loading buffer and sit in room temperature for 15 min before being analyzed by western blotting.

### Nucleus-cytoplasm extraction

MDA-MB-231 and HCC1806 cells were cultured in 6 cm dished until reached 70-80% confluence. Cells were then digested and washed with PBS. After centrifuging at 1000g in 4 °C for 5 min, the cell pellets were lyses with cytoplasm lysis buffer (85 mM KCl, 0.5%vol NP-40, 5 mM PIPES, pH 8.0) with protease and phosphatase inhibitor cocktail for 10 min on ice with frequent shake. The lysis was centrifuged at 5000g for 10 min at 4 °C. The supernatant containing cytoplasm ingredient was collected and mixed with 4×SDS loading buffer. The nucleus pellets were washed with cytoplasm lysis buffer and centrifuged at 5000g for another 10 min at 4 °C. The pellets containing cell nucleus were lysed by 1×SDS loading buffer. Both cytoplasm and cytoplasm lysis with SDS loading buffer were sit in room temperature for 15 min before western blotting.

### Subcellular fraction isolation

Differential centrifugation was performed to isolate subcellular fraction as described 41. In brief, the cells were scraped and washed in cold PBS. The washed cells were resuspended with Buffer A (250 mM sucrose, 250 mM KCl, 5 mM MgCl_2_, 50 mM Tris-HCl, pH 7.4) and lysed by Dounce's homogenizer. The cell lysis was centrifuged at 1000g for 10 min at 4 °C to remove un-lysed cells and nucleus. Then the cytosolic suspension was centrifuged at 15000g for 10 minutes at 4 °C to pellet down the mitochondria and mitochondria-associated ER Membrane (MAM). The supernatant was furtherly centrifuged at 80000g for 3 h at 4 °C to pellet the ER fraction. The total lysate, mitochondrial (with MAM), ER, and cytosolic fractions were mixed with 4×SDS loading buffer and analyzed by western blotting with anti-CST1 or anti-MAGT1 and representative markers antibodies (Mitochondria, AIF; ER, Calnexin; Cytosol, GAPDH).

### Protein localization analysis by fluorescent protein

To generate ER targeted fluorescent protein, ER localization signals same as ER-MagFRET were added to the mTagBFP2 gene. And the mitochondrial targeting sequence same as Mito-MagFRET was added to mRuby3 gene to generate mitochondrial targeted RFP. The CST1 and MAGT1 genes were tagged with mEGFP and mScarlet-I3 to the C-terminal respectively as both proteins possessed N-terminal signal peptides. MDA-MB-231 and HCC1806 cells were transiently transfected for 48 h with ER-mTagBFP2, Mito-mRuby3 and CST1-mEGFP, or ER-mTagBFP2, CST1-mEGFP and MAGT1-mScarlet-I3. Then the cells were seeded into the 6-well plates with coverslips. The cells were fixed with 4% paraformaldehyde in 4 ℃ overnight After adhesion. The slips were sealed with an anti-fluorescence quencher. Images were acquired by Nikon AX R MP confocal microscope (Nikon, Japan) using 100x oil objective.

### Cellular thermal shift assays (CETSA)

MDA-MB-231 and HCC1806 cells were cultured in 10-cm dishes until reached 80-90% confluence. Cells were treated with 4 µM TAT, TAT-TRX2 or TAT-TM4_2_ for 4 h. After treatment, cells were digested by trypsin, and washed twice with PBS. After centrifugation, the cell pellets were resuspended in 360 µL of PBS with protease and phosphatase inhibitor cocktail, and aliquoted into six PCR tubes. These aliquots were subjected to various temperature gradients (ranging from 40.0 °C to 60.0 °C) for 3 minutes in an Eppendorf Mastercycler nexus gradient (Eppendorf, Germany), followed by incubation at room temperature for 3 minutes. Then the samples underwent three cycles of freeze-thawing in liquid nitrogen to obtain complete lysis of cells. The lysates were transferred to 1.5 mL microcentrifuge tubes and centrifuged at 15000g for 20 minutes at 4 °C. From each sample, 60 µL of supernatant was collected to a new 1.5 mL microcentrifuge tubes, mixed with 60 µL of 2×SDS loading buffer, and denatured at room temperature for 15 min. The samples were then analyzed by western blotting.

### *In vivo* tumorigenesis assays

Female nude mice (approximately 6 weeks old, Hunan SJA Laboratory Animal Co., Ltd., China) were housed in the Specific Pathogen Free (SPF) animal facility at the Kunming Institute of Zoology, Chinese Academy of Sciences. HCC1806 cells (2×10^6^ cells/spot) were injected bilaterally into the 4th pair of mouse mammary gland fat pads. When the tumor volume approached above 50 mm³ at 5 to 7 days post injection, the tumor size was measured with a vernier caliper and mouse body weight was recorded using a scale per day. For TATs injection, the mice were randomly assigned to three groups and administered the drug once every two days at dosage of 5 mg/kg. The weight of the mice and the tumor volume were recorded every two days throughout the experiment. At the end of the experiment, the mice were euthanized, and tumor mass was measured. Tumor tissue samples were collected for further analysis, including immunohistochemical analysis. To evaluate potential compound toxicity, blood was collected via retro-orbital puncture from anesthetized mice at the study endpoint. Mouse serum was obtained by centrifugation at 4000g for 20 min. Aspartate aminotransferase (AST) and alanine aminotransferase (ALT) levels in serum was analyzed using commercial kits (Nanjing Jiancheng Bioengineering Institute, China) following the manufacturer's instructions. This animal experiment followed the ARRIVE guidelines and had been approved by the Animal Ethics Committee of the Kunming Medical University (IACUC-RE-2026-01-011).

### Immunohistochemical staining

Tumor tissues were fixed in 4% paraformaldehyde, embedded in paraffin, and sectioned at 4 μm thickness. Following deparaffinization and rehydration, antigen retrieval was performed using EDTA buffer (pH 8.0) at 95 °C for 15 minutes. Endogenous peroxidase activity was quenched with 3% H₂O₂ for 10 minutes. Sections were blocked with 5% BSA and then incubated overnight at 4 °C with anti-Ki67 (RMA-0542, MXB biotechnologies, China) primary antibody. After washing, the sections were incubated with HRP-conjugated secondary antibody for 1 hours at room temperature. Diaminobenzidine (DAB) was used as the chromogen, and hematoxylin was applied for counterstaining. Finally, sections were dehydrated, cleared, and mounted. Images were acquired using ZEISS Axio Scan.Z1 (Zeiss).

### Statistical analysis

Statistical analyses were conducted via Prism v.8.0.2 (GraphPad). The error bars indicate the mean ± standard error (SD). Two-way ANOVA or two-tailed Student's t test was employed to assess statistical significance. In the results, P < 0.05 was considered to be significant and marked with *, P < 0.01 was marked with ** and P < 0.001 was marked with ***, and P ≥ 0.05 was marked with ns, which means no significance.

## Supplementary Material

Supplementary figures.

Supplementary table 1.

## Figures and Tables

**Figure 1:**
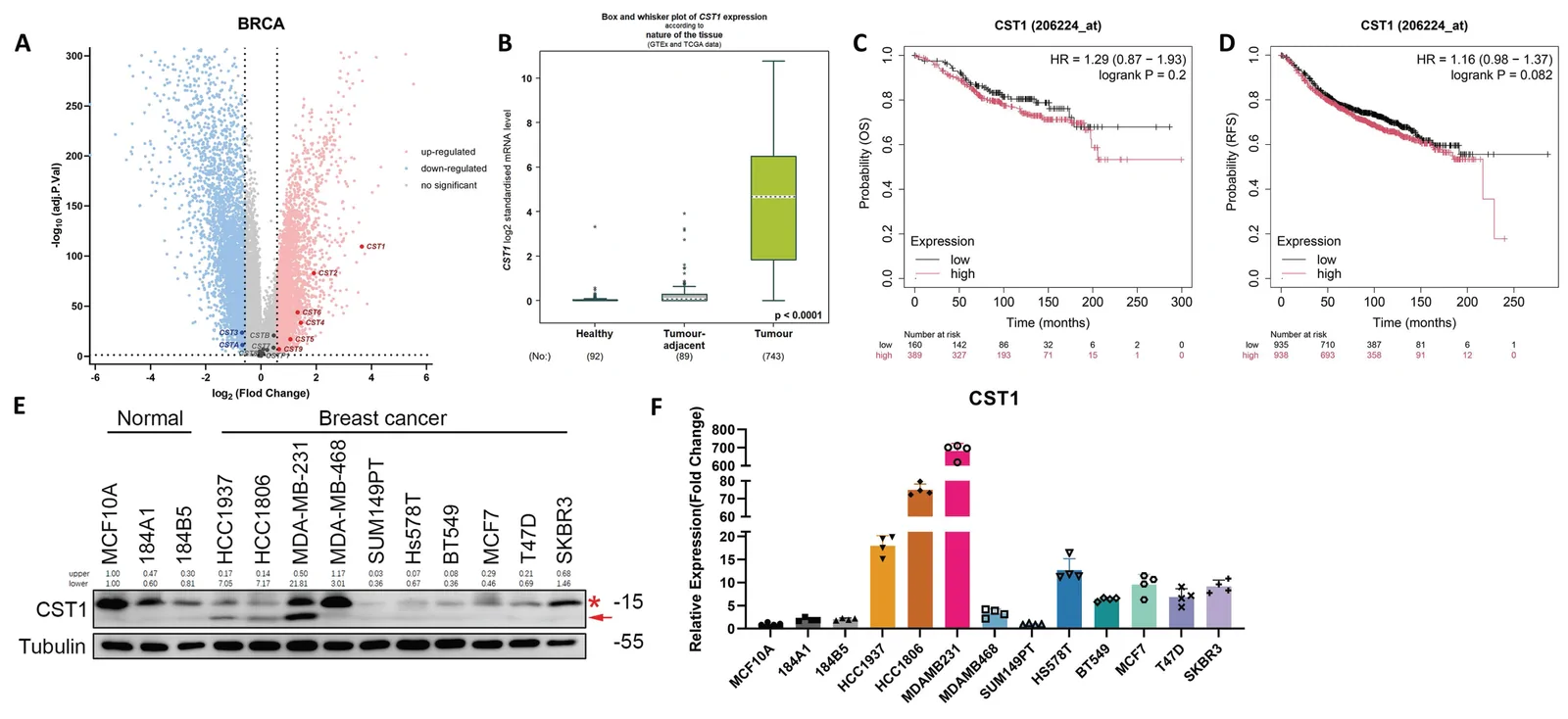
** CST1 was upregulated in breast cancer tissues and cell lines. A** Differential expression analysis using TCGA database. Genes with fold change over 1.5 and adj. P value below 0.05 are defined as significant different expressed. Genes that belong to Cystatin superfamily are highlighted. **B** Higher expression of *CST1* is found in tumor tissues compared with healthy or tumor-adjacent tissues using Breast Cancer Gene-Expression Miner v5.2 website (https://bcgenex.ico.unicancer.fr/BC-GEM/GEM-Accueil.php?js=1) with data sourced from TCGA database. **C, D** Aplan-Meier plots of overall survival and progression-free survival for breast cancer patients with systemically treatment using the Kaplan-Meier Plotter website based on TCGA database. **E, F** Western blotting and RT-qPCR analysis of CST1 expression in different breast cell lines. In WB, total Tubulin was used as a loading control. In RT-qPCR, total GAPDH was used as a loading control. In RT-qPCR, Data are presented as the mean ± SD (n=4).

**Figure 2:**
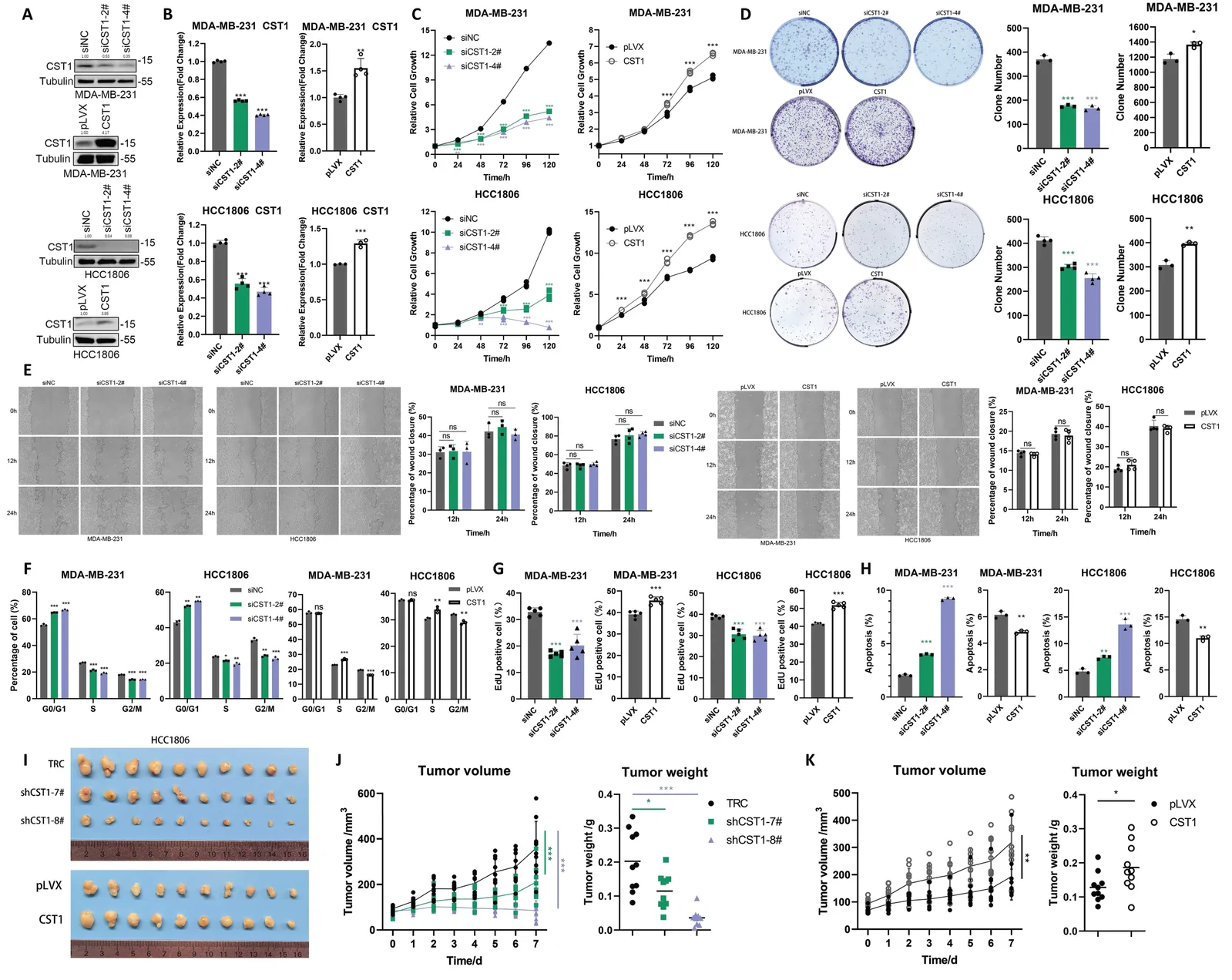
** CST1 promoted breast cancer proliferation both *in vitro* and *in vivo* but not metastasis. A, B** Western blotting and RT-qPCR analysis of CST1 knock-down and over-expression in breast cancer cell lines MDA-MB-231 and HCC1806. In WB, total Tubulin was used as a loading control. In RT-qPCR, total ACTB was used as a loading control. **C** SRB assay analyzed the proliferation of CST1 knock-down and over-expression MDA-MB-231 and HCC1806 cell lines. Data are presented as the mean ± SD (n=3 or 4). **D** Colony formation assay for assessing the proliferation capacity of CST1 knock-down and over-expression MDA-MB-231 and HCC1806 cell lines. Data are presented as the mean ± SD (n=3). **E** Wound healing analysis for assessing the migration of CST1 knock-down and over-expression MDA-MB-231 and HCC1806 cell lines. Representative images and quantification are shown. Data are presented as the mean ± SD (n=3 or 4). **F** Cell cycle analysis of CST1 knock-down and over-expression MDA-MB-231 and HCC1806 cell lines by flow cytometry using PI staining. Data are presented as the mean ± SD (n=3). **G** EdU assay for assessing the DNA synthesis capacity of CST1 knock-down and over-expression MDA-MB-231 and HCC1806 cell lines. Data are presented as the mean ± SD (n=5). **H** Cell apoptosis analysis of CST1 knock-down and over-expression MDA-MB-231 and HCC1806 cell lines by flow cytometry using PI and Annexin V-FITC staining. Data are presented as the mean ± SD (n=3). **I** Images of the xenograft tumors of CST1 stable knock-down and over-expression HCC1806 cells in nude mice. **J** Tumor volume and tumor weights changes of the xenograft tumors of CST1 stable knock-down HCC1806 cells in nude mice. Tumor volume was calculated as length timed width squared and divided by 2. Data are presented as the mean ± SD (n=10). **K** Tumor volume and tumor weights changes of the xenograft tumors of CST1 stable over-expression HCC1806 cells in nude mice. Data are presented as the mean ± SD (n=10).

**Figure 3:**
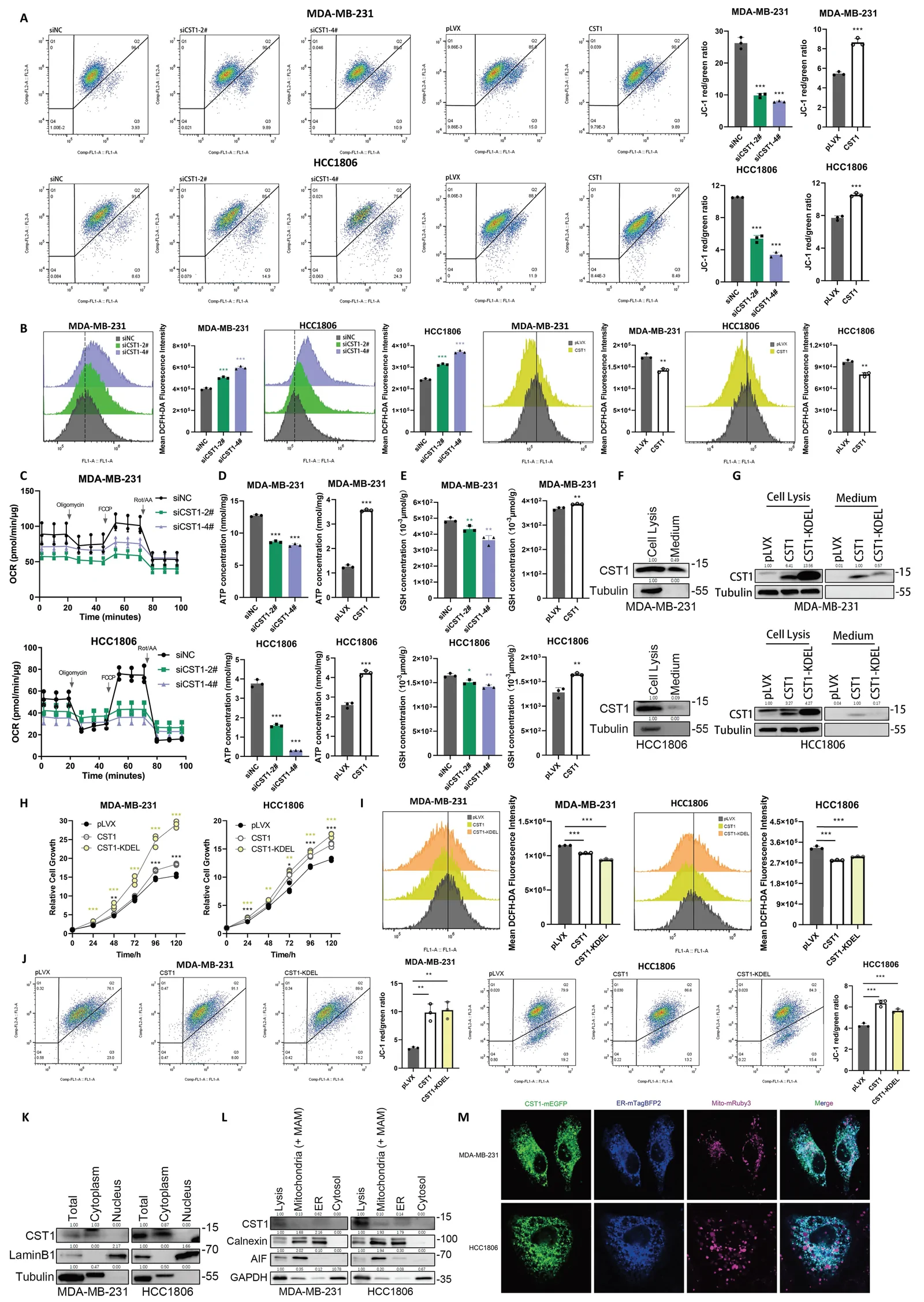
** CST1 promoted tumorigenesis by regulating mitochondria metabolism in a secretion independent manner. A** Mitochondrial membrane potential was determined in CST1 knock-down and over-expression MDA-MB-231 and HCC1806 cell lines by flow cytometry using JC-1 staining. Data are presented as the mean ± SD (n=3). **B** Intracellular ROS level was determined in CST1 knock-down and over-expression MDA-MB-231 and HCC1806 cell lines by flow cytometry using DCFH-DA staining. Data are presented as the mean ± SD (n=3). **C** The OCR levels were measured in MDA-MB-231 and HCC1806-CST1 knock-down cell lines. Data are presented as the mean ± SD (n=3). **D** Intracellular ATP level was determined in MDA-MB-231 and HCC1806-CST1 knock-down and over-expression cell lines. Data are presented as the mean ± SD (n=3). **E** Intracellular GSH level was determined in CST1 knock-down and over-expression MDA-MB-231 and HCC1806 cell lines. Data are presented as the mean ± SD (n=3). **F** Western blotting analysis of CST1 secretion in breast cancer cell lines MDA-MB-231 and HCC1806. **G** Western blotting analysis of CST1 and CST1-KDEL over-expression level and secretion in breast cancer cell lines MDA-MB-231 and HCC1806. **H** SRB assay analyzed the proliferation of CST1 and CST1-KDEL over-expression MDA-MB-231 and HCC1806 cell lines. Data are presented as the mean ± SD (n=3). **I** Intracellular ROS level was determined in CST1 and CST1-KDEL over-expression MDA-MB-231 and HCC1806 cell lines by flow cytometry using DCFH-DA staining. Data are presented as the mean ± SD (n=3). **J** Mitochondrial membrane potential was determined in CST1 and CST1-KDEL over-expression MDA-MB-231 and HCC1806 cell lines by flow cytometry using JC-1 staining. Data are presented as the mean ± SD (n=3). **K** Nucleus-cytoplasm extraction assay followed by Western blotting analysis of CST1 was performed in MDA-MB-231 and HCC1806 cell lines. The Tubulin was used as a cytoplasm marker; the LaminB1 was used as a nucleus marker. **L** Subcellular fractions of MDA-MB-231 and HCC1806 cell lines were subjected to western blot analysis. The GAPDH was used as a cytoplasm marker; the AIF was used as a mitochondria marker; the Calnexin was used as an ER marker. **M** Confocal immunofluorescence of CST1-mEGFP, ER-mTagBFP2 and Mito-mRuby3 in MDA-MB-231 and HCC1806 cell lines.

**Figure 4:**
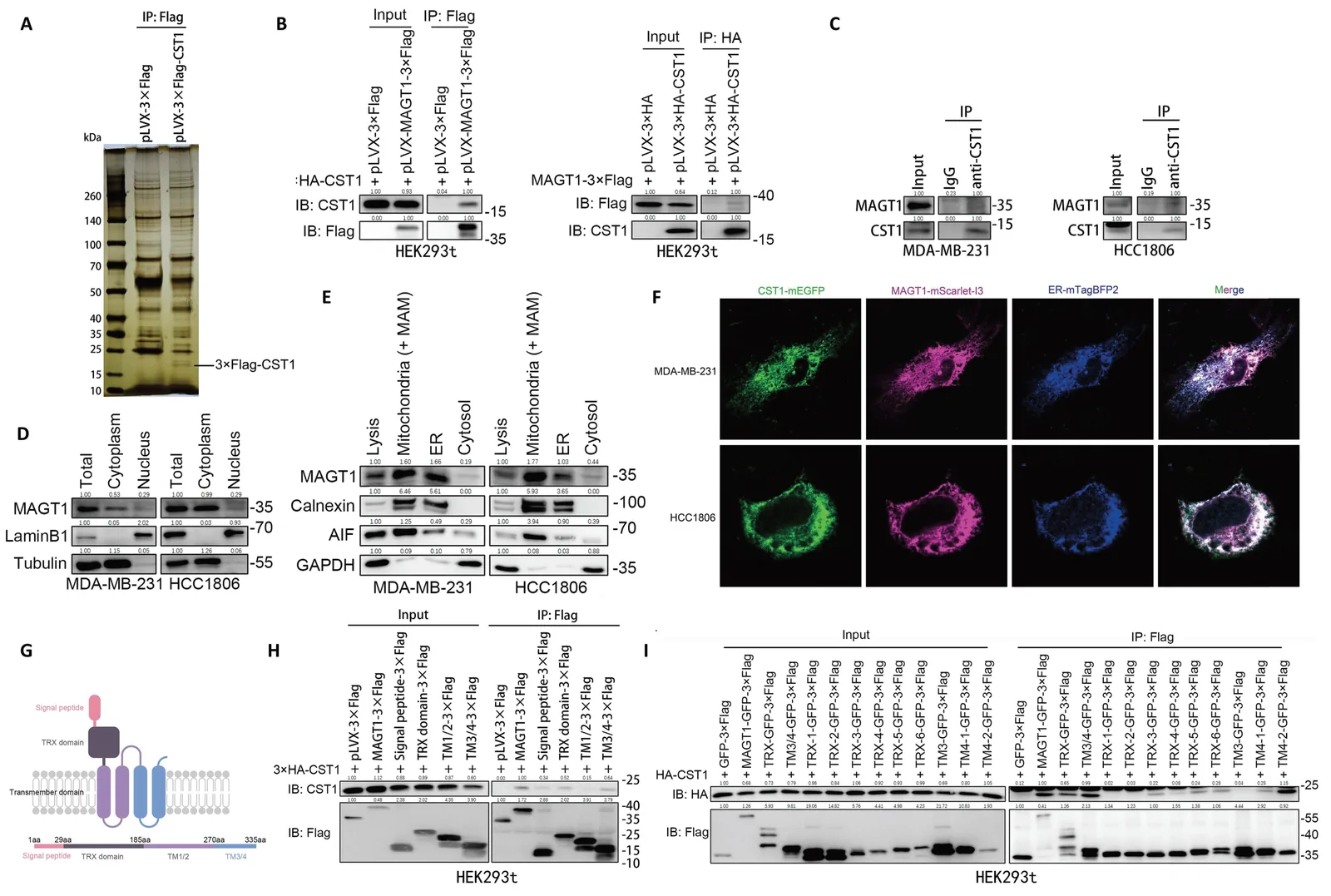
** MAGT1 interacted with CST1 through multiply domains on ER membrane. A** Sliver stain of co-IP lysis. The lane of CST1 is marked on the gel image. **B** Exogenous co-IP of 3×HA-CST1 and MAGT1-3×Flag by anti-Flag or anti-HA magnetic beads in HEK293t cells. **C** Endogenous co-IP of CST1 and MAGT1 by anti-CST1 antibody and protein A/G magnetic beads in MDA-MB-231 and HCC1806 cells. **D** Nucleus-cytoplasm extraction assay followed by Western blotting analysis of MAGT1 was performed in MDA-MB-231 and HCC1806 cell lines. The Tubulin was used as a cytoplasm marker; the LaminB1 was used as a nucleus marker. **E** Subcellular fractions of MDA-MB-231 and HCC1806 cell lines were subjected to western blot analysis. The GAPDH was used as a cytoplasm marker; the AIF was used as a mitochondria marker; the Calnexin was used as an ER marker. **F** Confocal immunofluorescence of CST1-mEGFP, MAGT1-mScarlet-I3 and ER-mTagBFP2 in MDA-MB-231 and HCC1806 cell lines. **G** Predicted structure of MAGT1. **H, I** Exogenous co-IP analysis of 3×HA-CST1 and N-terminal 3×Flag or GFP-3×Flag tagged different MAGT1 domains by anti-Flag magnetic beads in HEK293t cells.

**Figure 5:**
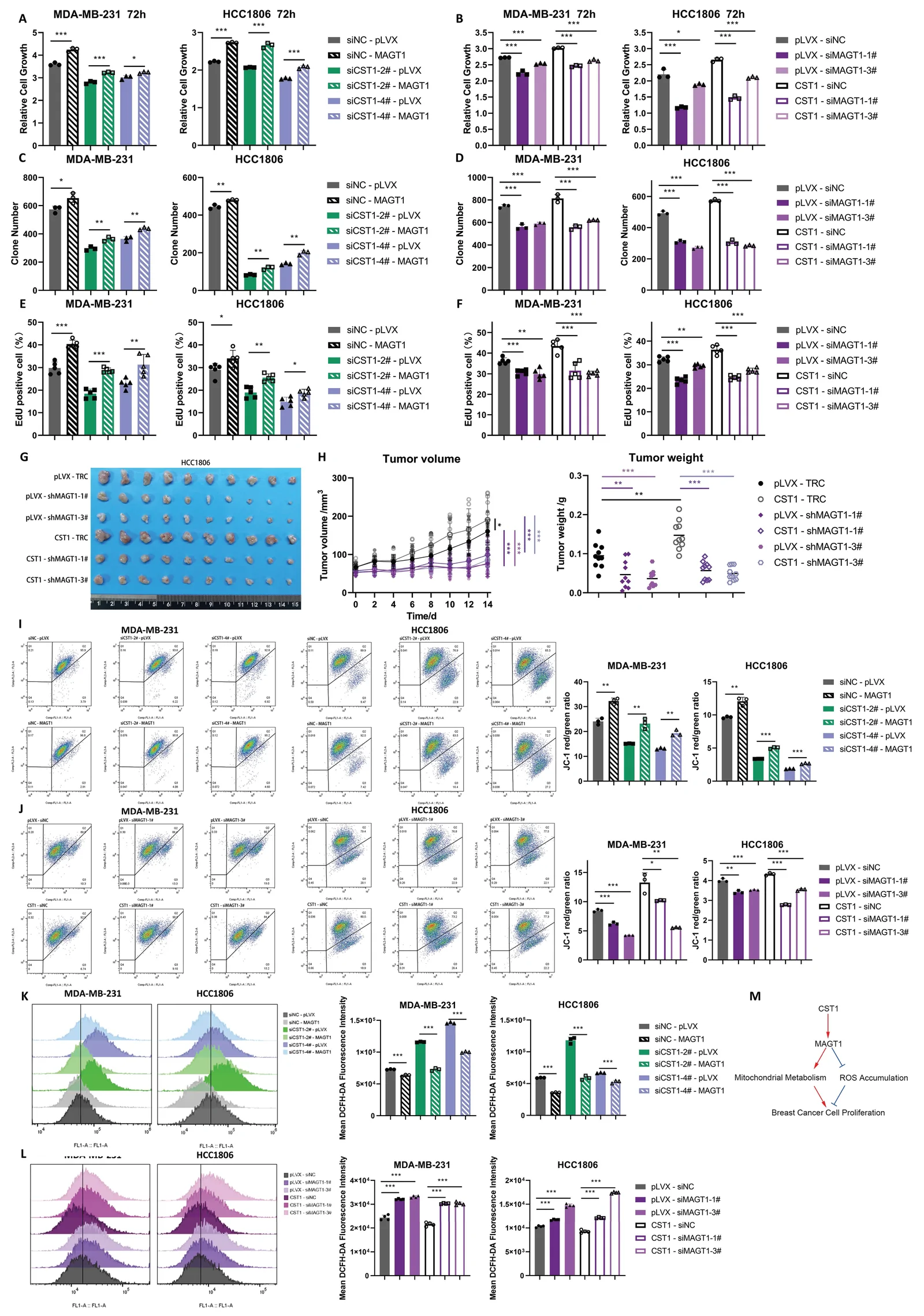
** CST1 functions through MAGT1 in breast cancer. A** SRB assay analyzed the rescue effects in proliferation of MAGT1 over-expression in MDA-MB-231 and HCC1806-CST1 knock-down cell lines. Data are presented as the mean ± SD (n=3). **B** SRB assay analyzed the rescue effects in proliferation of MAGT1 knock-down in MDA-MB-231 and HCC1806-CST1 over-expression cell lines. Data are presented as the mean ± SD (n=3). **C** Colony formation assay for assessing the rescue effects in proliferation of MAGT1 over-expression in MDA-MB-231 and HCC1806-CST1 knock-down cell lines. Data are presented as the mean ± SD (n=3). **D** Colony formation assay for assessing the rescue effects in proliferation of MAGT1 knock-down in MDA-MB-231 and HCC1806-CST1 over-expression cell lines. Data are presented as the mean ± SD (n=3). **E** EdU assay for assessing the rescue effects in proliferation of MAGT1 over-expression in MDA-MB-231 and HCC1806-CST1 knock-down cell lines. Data are presented as the mean ± SD (n=3). **F** EdU assay for assessing the rescue effects in proliferation of MAGT1 knock-down in MDA-MB-231 and HCC1806-CST1 over-expression cell lines. Data are presented as the mean ± SD (n=3). **G** Images of the xenograft tumors of MAGT1 over-expression and CST1 knock-down HCC1806 cells in nude mice. **H** Tumor volume and tumor weights changes of the xenograft tumors of MAGT1 over-expression and CST1 knock-down HCC1806 cells in nude mice. Tumor volume was calculated as length timed width squared and divided by 2. Data are presented as the mean ± SD (n=10). **I** Mitochondrial membrane potential assay analyzed the rescue effects of MAGT1 over-expression in MDA-MB-231 and HCC1806-CST1 knock-down cell lines by flow cytometry. Data are presented as the mean ± SD (n=3). **J** Mitochondrial membrane potential assay analyzed the rescue effects of MAGT1 knock-down in MDA-MB-231 and HCC1806-CST1 over-expression cell lines by flow cytometry. Data are presented as the mean ± SD (n=3). **K** Rescue effects in intracellular ROS level of MAGT1 over-expression in MDA-MB-231 and HCC1806-CST1 knock-down cell lines by flow cytometry. Data are presented as the mean ± SD (n=3). **L** Rescue effects in intracellular ROS level of MAGT1 knock-down in MDA-MB-231 and HCC1806-CST1 over-expression cell lines by flow cytometry. Data are presented as the mean ± SD (n=3). **M** A summary of this section, CST1 promoted breast cancer cell proliferation through MAGT1 mediated mitochondrial metabolism and ROS clearance.

**Figure 6:**
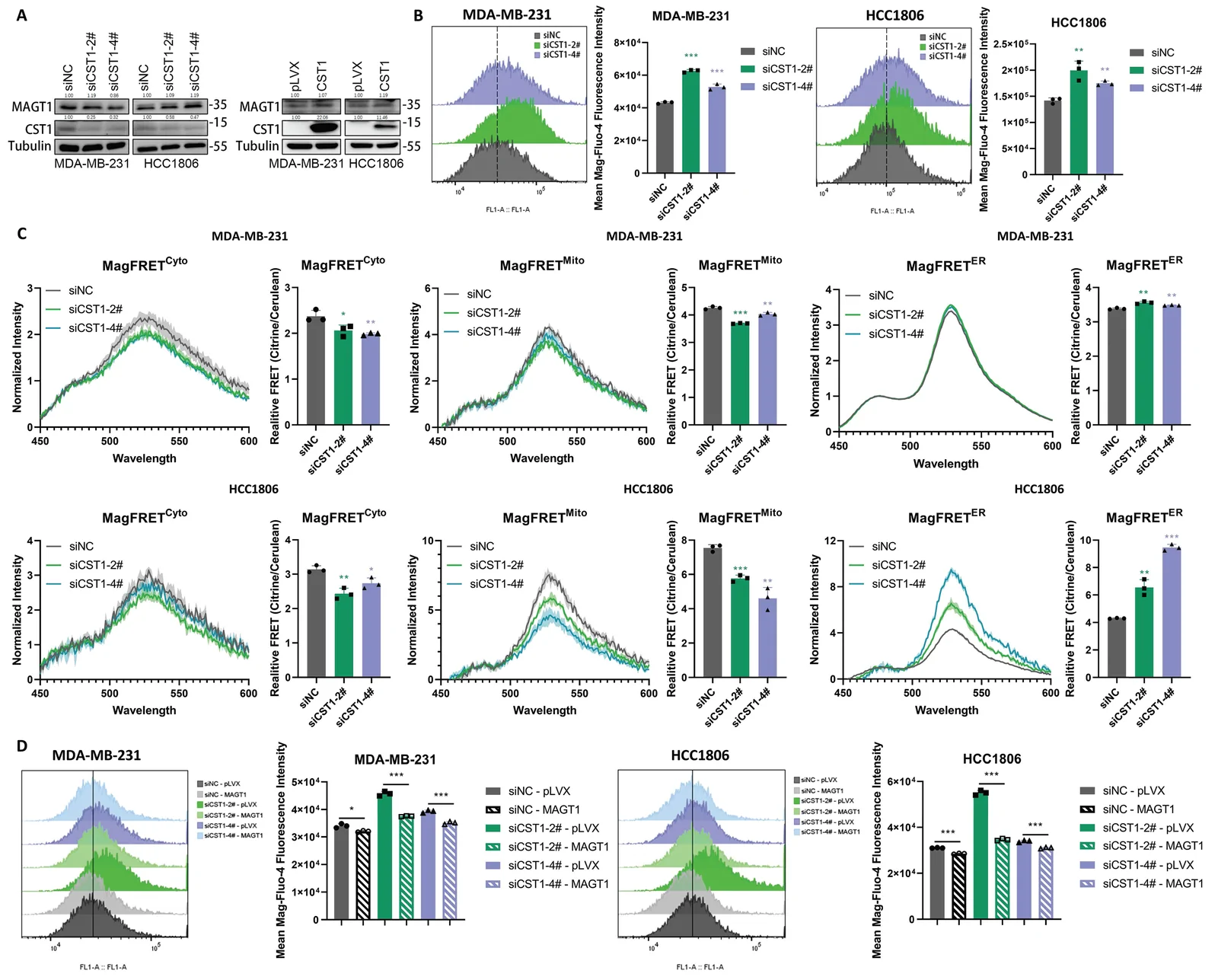
** CST1 regulated the magnesium transport ability of MAGT1 without altering MAGT1 expression. A** Western blotting analysis of MAGT1 expression in MDA-MB-231 and HCC1806-CST1 knock-down and over-expression cell lines. Total Tubulin was used as a loading control. **B** Intracellular magnesium level in MDA-MB-231 and HCC1806-CST1 knock-down cells by flow cytometry using Mag-fluo-4-AM staining. Data are presented as the mean ± SD (n=3). **C** Magnesium level in cytoplasm, mitochondria and ER in MDA-MB-231 and HCC1806-CST1 knock-down cells detected by MagFRET. Data are presented as the mean ± SD (n=3). **D** Rescue effects in intracellular magnesium level of MAGT1 over-expression in MDA-MB-231 and HCC1806-CST1 knock-down cell lines by flow cytometry. Data are presented as the mean ± SD (n=3).

**Figure 7:**
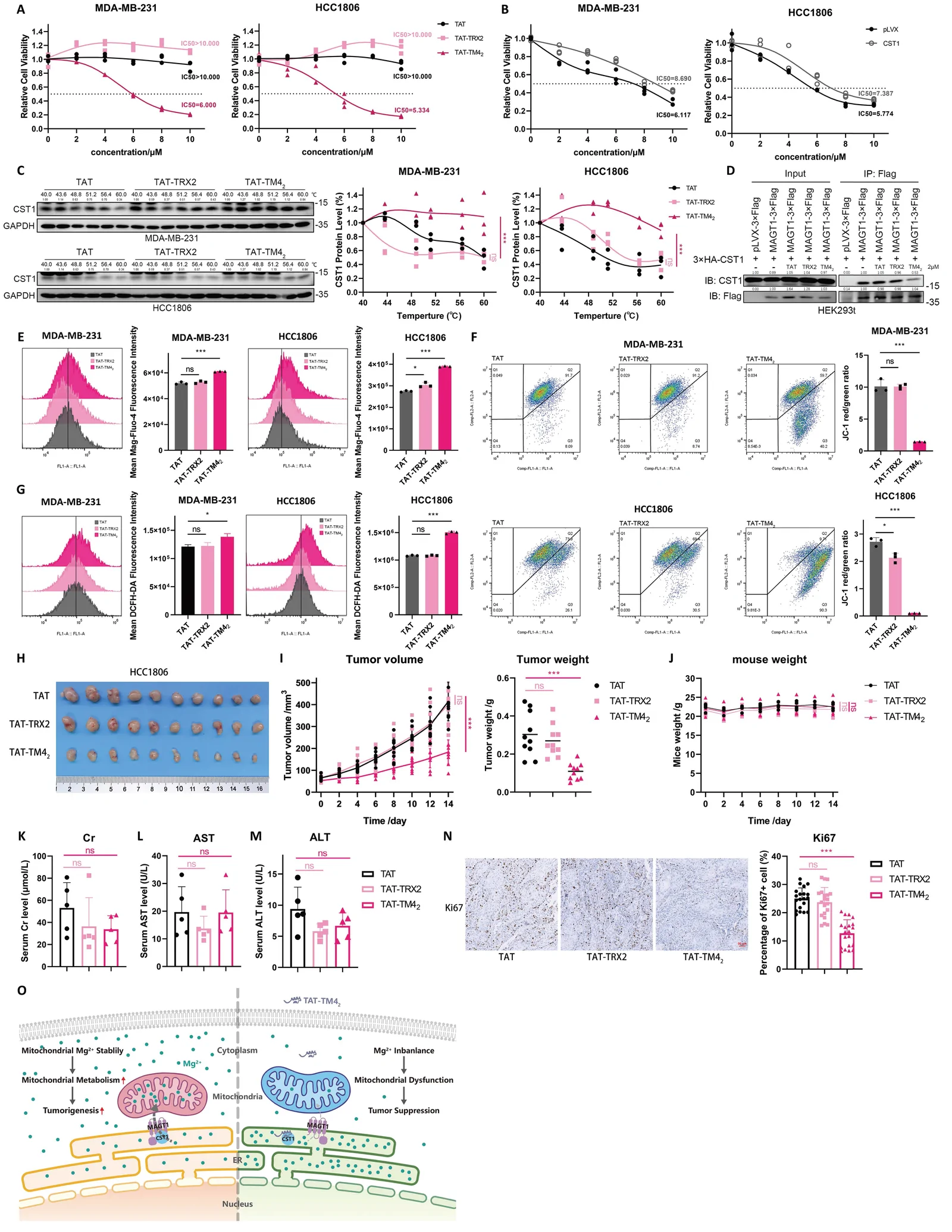
** Inhibiting CST1-MAGT1 interaction by a CCP contributed to anti-tumor therapy *in vitro* and *in vivo*. A** CCK8 assay analyzed the cell viability of MDA-MB-231 and HCC1806 cell lines after treatment with three CCPs TAT, TAT-TRX2 and TAT-TM4_2_. Data are presented as the mean ± SD (n=3). **B** CCK8 assay analyzed the cell viability of CST1 over-expression MDA-MB-231 and HCC1806 cell lines after treatment with TAT-TM4_2_. Data are presented as the mean ± SD (n=3). **C** CETSA analyzed the binding between CCPs and CST1. Total GAPDH was used as a loading control. Data are presented as the mean ± SD (n=3). **D** immunoprecipitation assay analyzed that TAT-TM4_2_ disrupted the binding between CST1 and MAGT1. **E** Intracellular magnesium level in MDA-MB-231 and HCC1806 cell lines treated with CCPs determined by flow cytometry using Mag-fluo-4-AM staining. Data are presented as the mean ± SD (n=3). **F** Mitochondrial membrane potential in MDA-MB-231 and HCC1806 cell lines treated with CCPs by flow cytometry. Data are presented as the mean ± SD (n=3). **G** Intracellular ROS level in MDA-MB-231 and HCC1806 cell lines treated with CCPs by flow cytometry. Data are presented as the mean ± SD (n=3). **H** Images of the xenograft tumors of HCC1806 cells in nude mice treated with CCPs. **I** Tumor volume and tumor weights changes of the xenograft tumors of HCC1806 cells in nude mice treated with CCPs. Tumor volume was calculated as length timed width squared and divided by 2. Data are presented as the mean ± SD (n=10). **J** Mouse weight weights changes of the nude mice treated with CCPs. Data are presented as the mean ± SD (n=5). **K-M** The levels of creatinine (Cr), aspartate aminotransferase (AST) and alanine aminotransferase (ALT) in mouse serum of the nude mice treated with CCPs. Data are presented as the mean ± SD (n=5). **N** Immunohistochemistry (IHC) analysis and quantification of Ki67-positive cells in tumor tissues (brown). Immunohistochemistry slides were randomly selected for quantitative analysis of positive staining using standardized scoring criteria by imageJ. Scale bar: 50 µm. Data are presented as mean ± SD (*n* = 20). **O** Model of this work. CST1 interacted with MAGT1 and promoted the Mg^2+^ transporting ability of MAGT1 on endoplasmic reticulum membrane, thereby maintaining the Mg^2+^ concentration in mitochondria and promoting mitochondria metabolism. A cell-penetrating peptide (CCP), TAT-TM4_2_ had antitumor effect by inhibiting CST1-MAGT1 interaction in breast cancer cells.
