# Supplementary material for: CST1 Regulates ER-Mitochondrial Mg²⁺ Balance via MAGT1 to Promote Breast Cancer Metabolism and Growth
Source: Int J Biol Sci. 2026 Sep 11;22(15):8230–50. doi: 10.7150/ijbs.136015 (PMC13685611; doi:10.7150/ijbs.136015)
Supplement: Supplementary file 1 [file ijbsv22p8230s1.pdf]

Supplementary figures

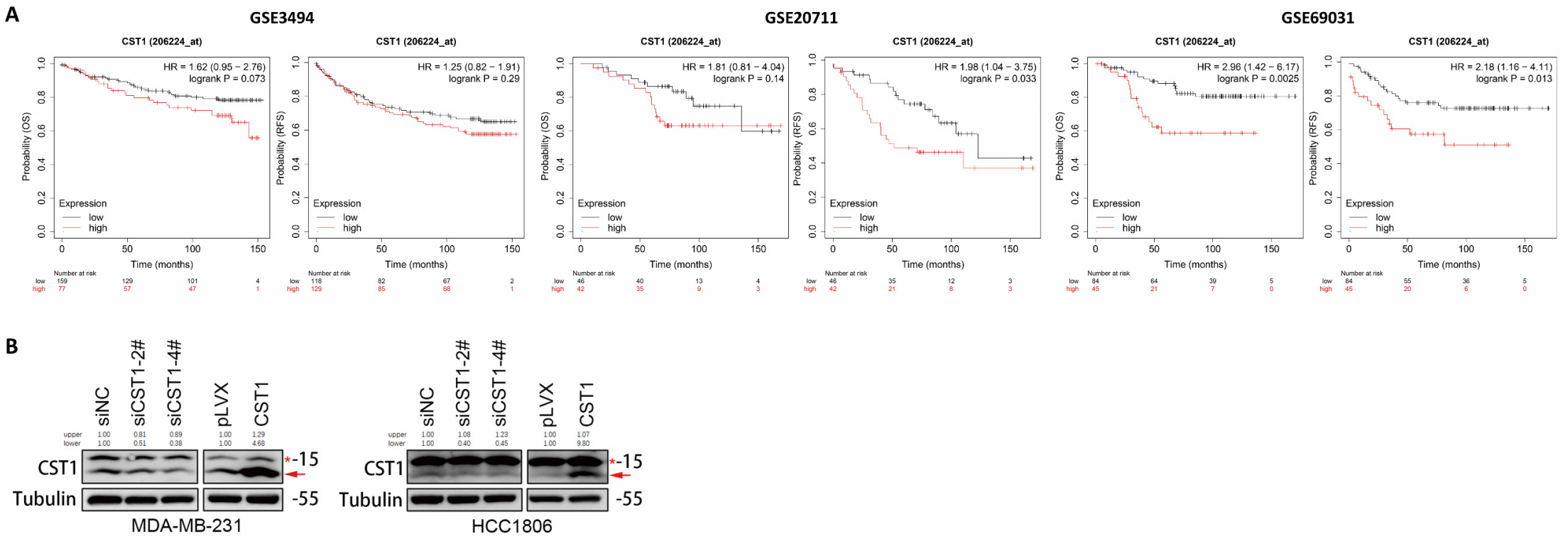

Fig. S1 Expression level of CST1 was negatively corelated with worse clinic outcomes.

A Aplan–Meier plots of overall survival and progression-free survival for breast cancer patients using the Kaplan-Meier Plotter website based on based on GEO GSE3494, GSE20711, GSE69031 databases.

B Western blotting analysis of CST1 knock-down and over-expression in breast cancer cell lines MDA-MB-231 and HCC1806 with both two bands presented. Total Tubulin was used as a loading control.

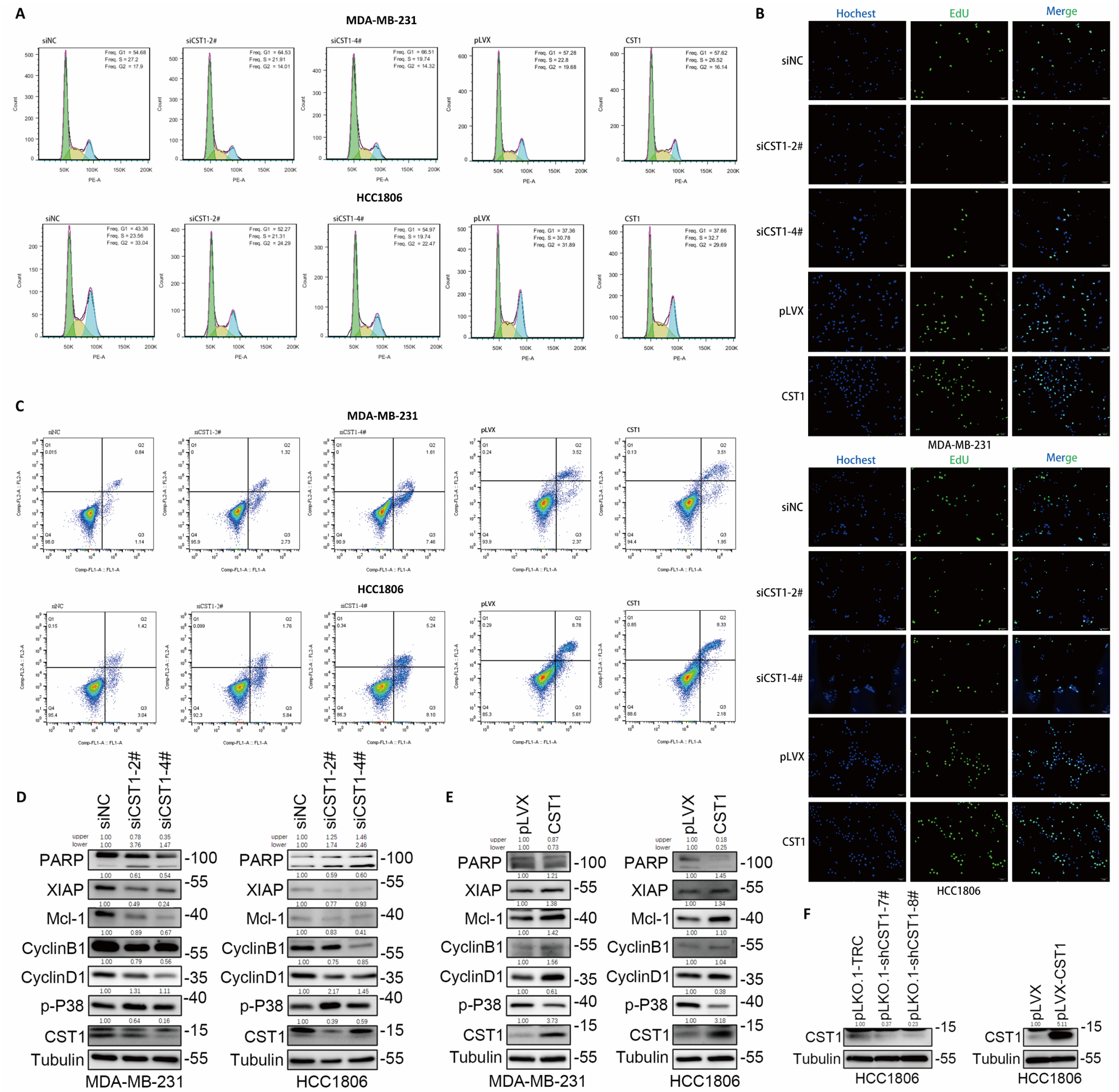

Fig. S2 CST1 promoted breast cancer proliferation both *in vitro* and *in vivo*.

A Imagines of cell cycle analysis of CST1 knock-down and over-expression MDA-MB-231 and HCC1806 cell lines by flow cytometry.

B Imagines of EdU assay for assessing the DNA synthesis capacity of CST1 knock-down and over-expression MDA-MB-231 and HCC1806 cell lines.

C Imagines of cell apoptosis analysis of CST1 knock-down and over-expression MDA-MB-231 and HCC1806 cell lines by flow cytometry.

D, E Western blotting for analyzing the expression of proteins associated with cell cycle and apoptosis in CST1 knock-down and over-expression MDA-MB-231 and HCC1806 cell lines. Total Tubulin was used as a loading control.

F Western blotting for confirming the expression changes of CST1 in stable knock-down and over-expression HCC1806 cells subsequently used in xenograft tumor assay.

Total Tubulin was used as a loading control.

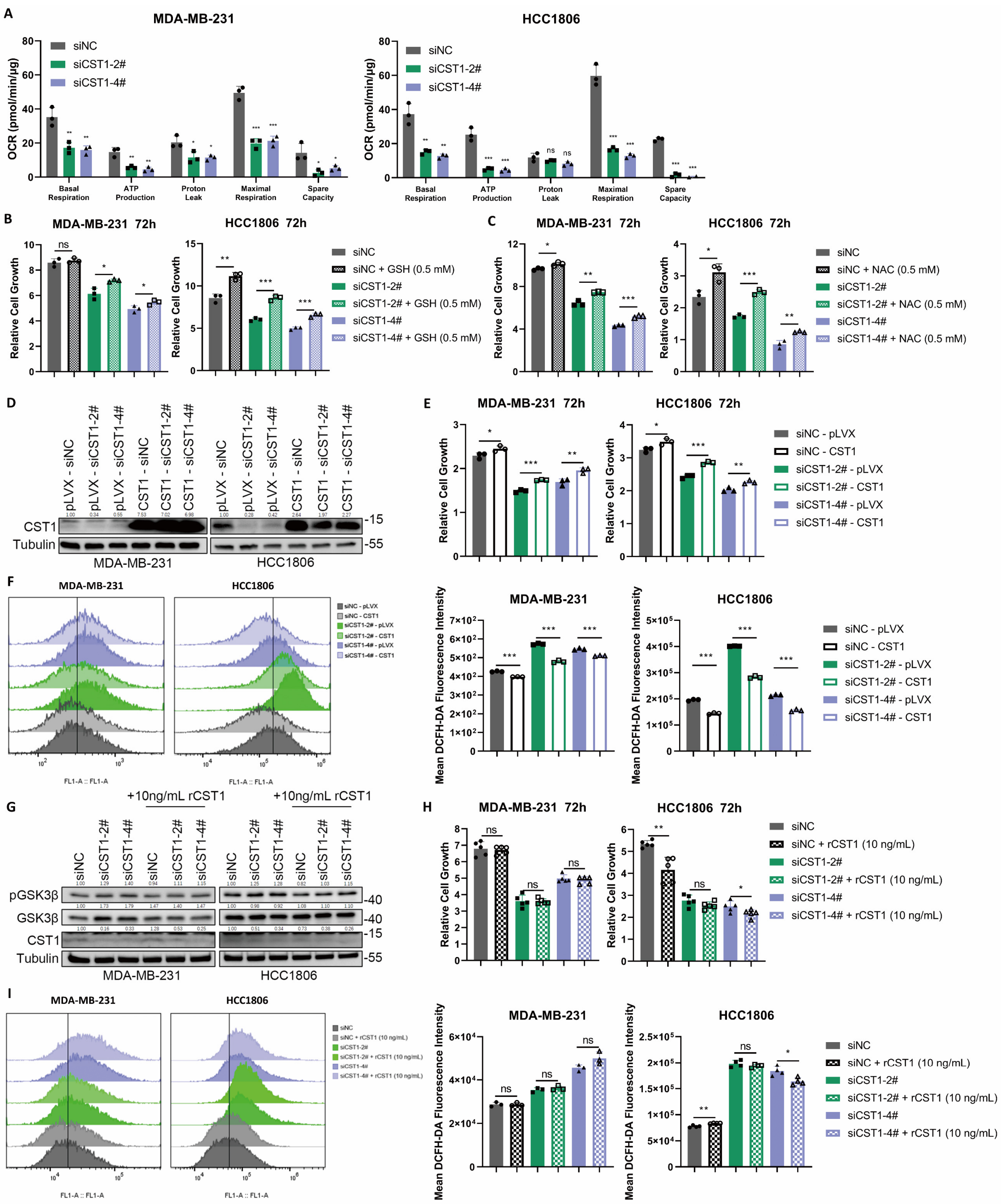

Fig. S3 CST1 promoted tumorigenesis in a secretion independent manner.

A Bar graphs of basal respiration, ATP production, proton leak, maximal respiration and spare capacity in the OCR assay. Data are presented as the mean ± SD (n=3).

B, C MDA-MB-231 and HCC1806-CST1 knock-down cell lines were treated with GSH (0.5 mmol/L) or NAC (0.5 mmol/L), and cell viability was detected via the SRB assay.

Data are presented as the mean ± SD (n=3).

D Western blotting analysis of CST1 over-expression in MDA-MB-231 and HCC1806-CST1 knock-down cell lines. Total Tubulin was used as a loading control.

E SRB assay analyzed the rescue effects in proliferation of CST1 over-expression in MDA-MB-231 and HCC1806-CST1 knock-down cell lines. Data are presented as the mean ± SD (n=3).

F The rescue effects in intracellular ROS level of CST1 over-expression in MDA-MB-231 and HCC1806-CST1 knock-down cell lines by flow cytometry. Data are presented as the mean ± SD (n=3).

G Western blotting analysis of relative proteins in rCST1 (10 ng/mL) treated MDA-MB-231 and HCC1806-CST1 knock-down cell lines. Total Tubulin was used as a loading control.

H SRB assay analyzed the rescue effects in proliferation of adding rCST1 (10 ng/mL) in MDA-MB-231 and HCC1806-CST1 knock-down cell lines. Data are presented as the mean ± SD (n=5).

I Rescue effects in intracellular ROS level of adding rCST1 (10 ng/mL) in MDA-MB-231 and HCC1806-CST1 knock-down cell lines by flow cytometry. Data are presented as the mean ± SD (n=3 or 4).

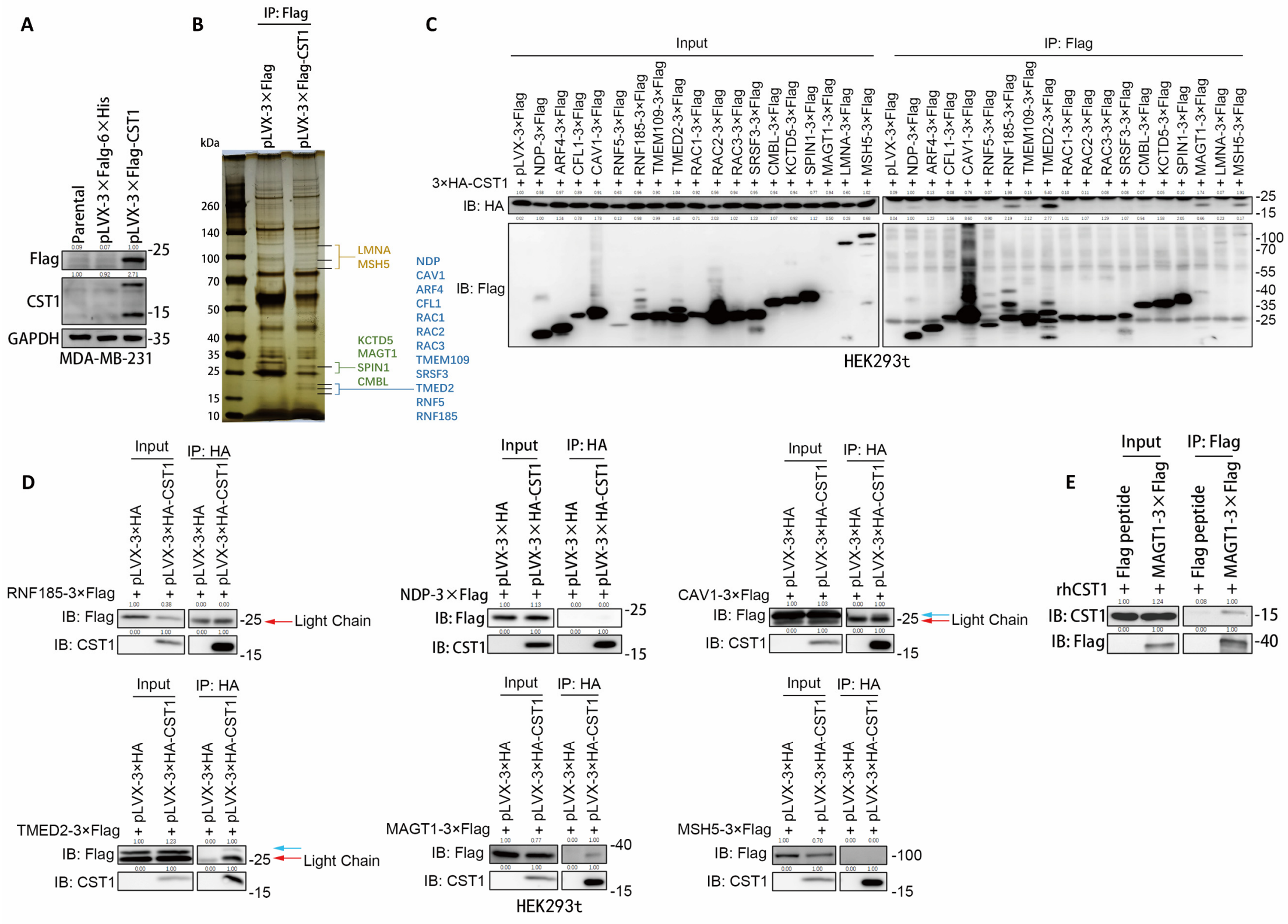

Fig. S4 CST1 interacted with MAGT1.

A Western blotting analysis of CST1-3×Flag over-expression in MDA-MB-231 cells.

B Sliver stain of co-IP lysis. Selected proteins are marked on the gel image.

C Exogenous co-IP of 3×HA-CST1 and selected proteins by anti-Flag magnetic beads in HEK293t cells.

D Exogenous co-IP of 3×HA-CST1 and six proteins observed to interact with CST1 in Fig **S4C** by anti-HA magnetic beads in HEK293t cells.

E Co-IP of purified MAGT1-3×Flag and rhCST1 proteins by Flag M2 beads.

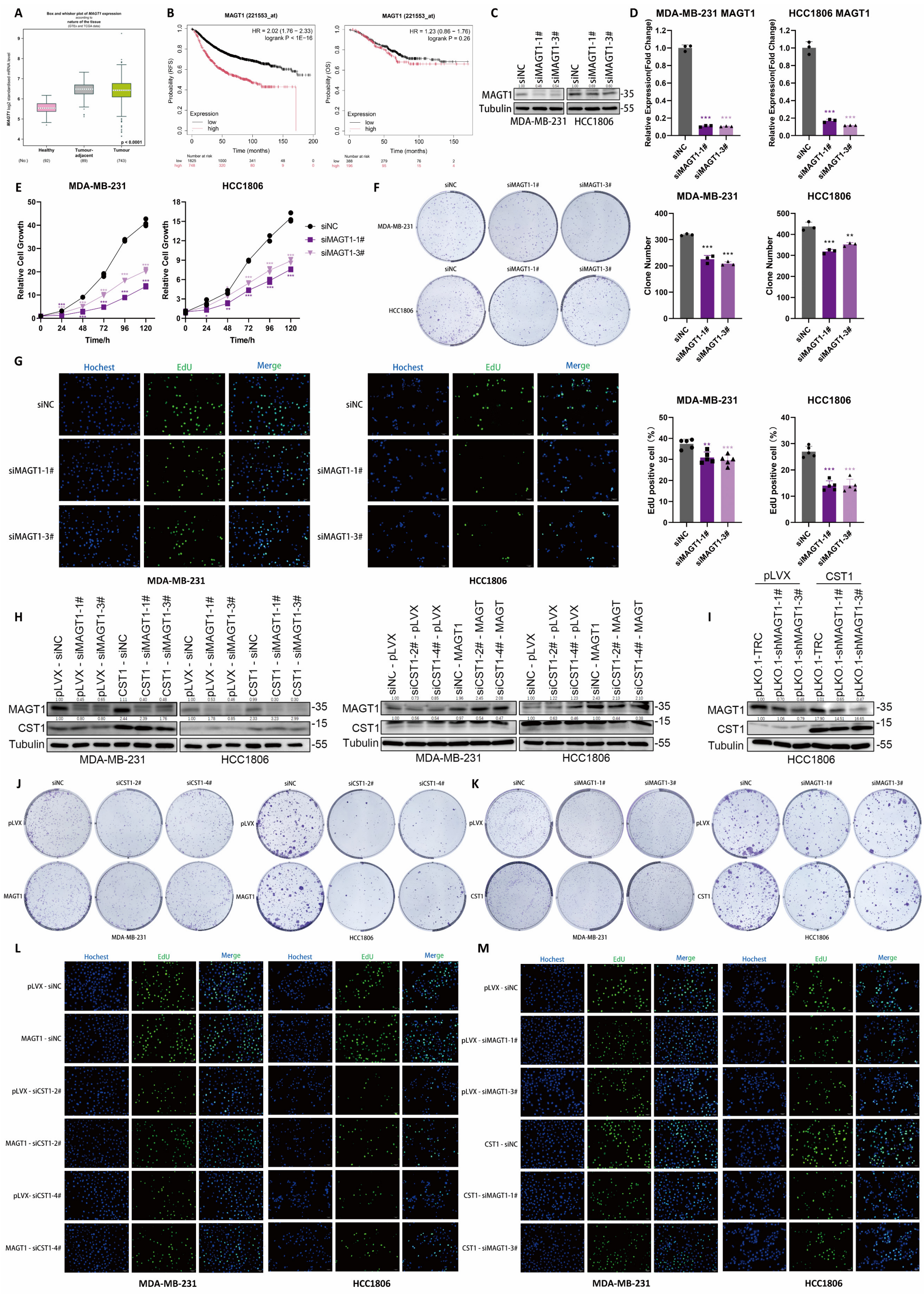

Fig. S5 MAGT1 was identified as an oncogene in breast cancer.

A Higher expression of *MAGT1* is found in tumor tissues compared with healthy tissues using Breast Cancer Gene-Expression Miner v5.2 website with data sourced from TCGA database.

B Aplan–Meier plots of overall survival and progression-free survival for breast cancer

patients using the Kaplan-Meier Plotter website based on TCGA database.

C, D Western blotting and RT-qPCR analysis of MAGT1 knock-down in breast cancer cell lines MDA-MB-231 and HCC1806. In WB, total Tubulin was used as a loading control. In RT-qPCR, total ACTB was used as a loading control.

E SRB assay analyzed the proliferation of MDA-MB-231 and HCC1806-MAGT1 knock-down cell lines. Data are presented as the mean ± SD (n=3).

F Colony formation assay for assessing the proliferation capacity of MDA-MB-231 and HCC1806-MAGT1 knock-down cell lines. Data are presented as the mean ± SD (n=3).

G EdU assay for assessing the DNA synthesis capacity of MDA-MB-231 and HCC1806-MAGT1 knock-down cell lines. Data are presented as the mean ± SD (n=5).

H Western blotting analysis of MAGT1 knock-down in MDA-MB-231 and HCC1806-CST1 over-expression cell lines, and MAGT1 over-expression in CST1 knock-down cell lines. Total Tubulin was used as a loading control.

I Western blotting analysis of MAGT1 stable knock-down in MDA-MB-231 and HCC1806-CST1 over-expression cell lines. Total Tubulin was used as a loading control.

J Images of the Colony formation assay for assessing the rescue effects in proliferation of MAGT1 over-expression in MDA-MB-231 and HCC1806-CST1 knock-down cell lines.

K Images of the Colony formation assay for assessing the rescue effects in proliferation of MAGT1 knock-down in MDA-MB-231 and HCC1806-CST1 over-expression cell lines.

L Images of the EdU assay for assessing the rescue effects in proliferation of MAGT1 over-expression in MDA-MB-231 and HCC1806-CST1 knock-down cell lines.

M Images of the EdU assay for assessing the rescue effects in proliferation of MAGT1 knock-down in MDA-MB-231 and HCC1806-CST1 over-expression cell lines.

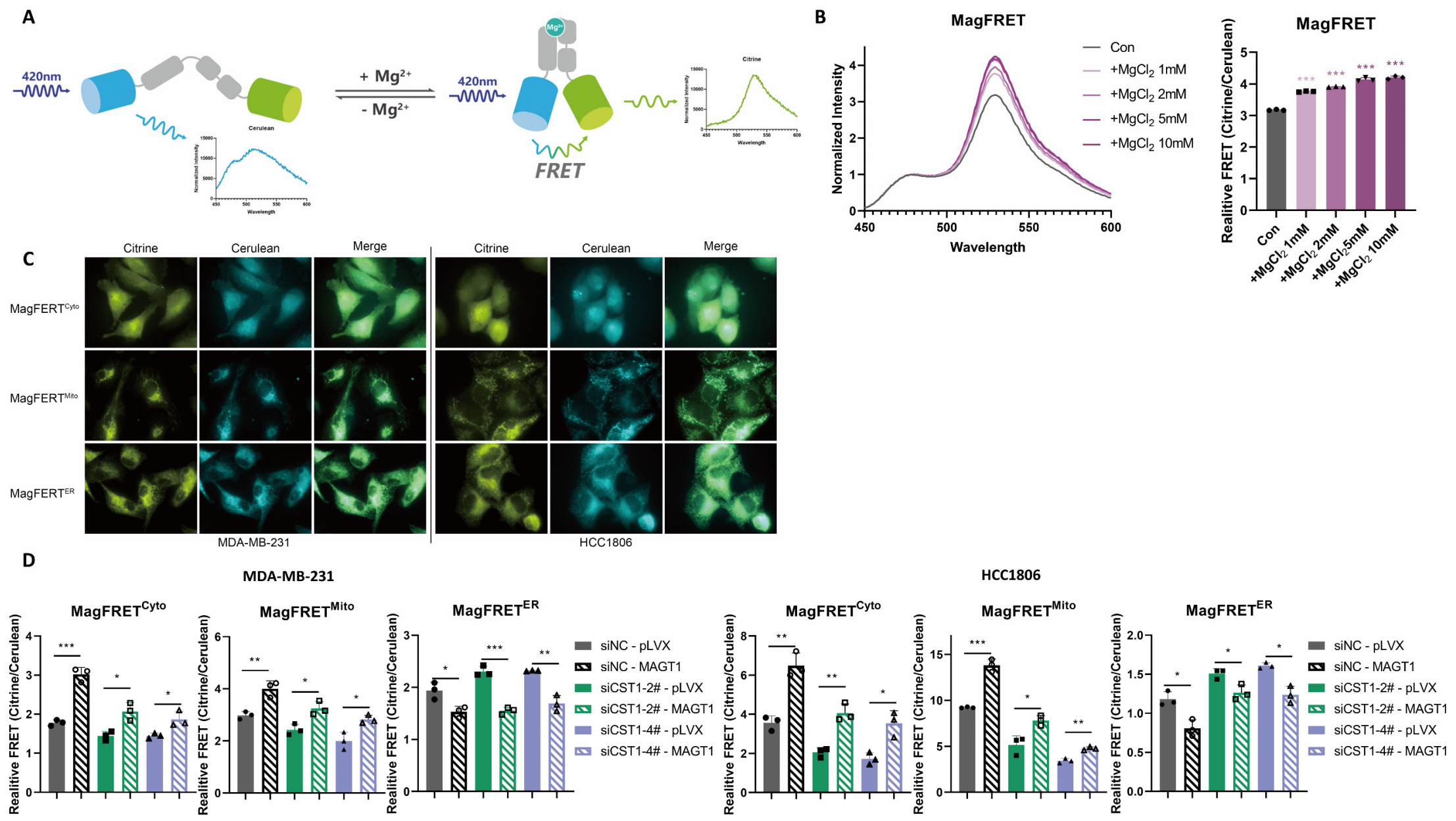

Fig. S6 Establishment of MagFRET^{Cyto}, MagFRET^{Mito} and MagFRET^{ER}.

A Model of MagFRET in magnesium level detection.

B FRET effects of MagFRET overexpression HEK293t cell lysis explosed to different Mg^{2+} level. Data are presented as the mean ± SD (n=3).

C Immunofluorescence of MagFRET^{Cyto}, MagFRET^{Mito} and MagFRET^{ER} in MDA-MB-231 and HCC1806 cell lines.

D Rescue effects in intracellular magnesium level in cytoplasm, mitochondria and ER of MAGT1 over-expression in MDA-MB-231 and HCC1806-CST1 knock-down cells detected by MagFRET. Data are presented as the mean ± SD (n=3).

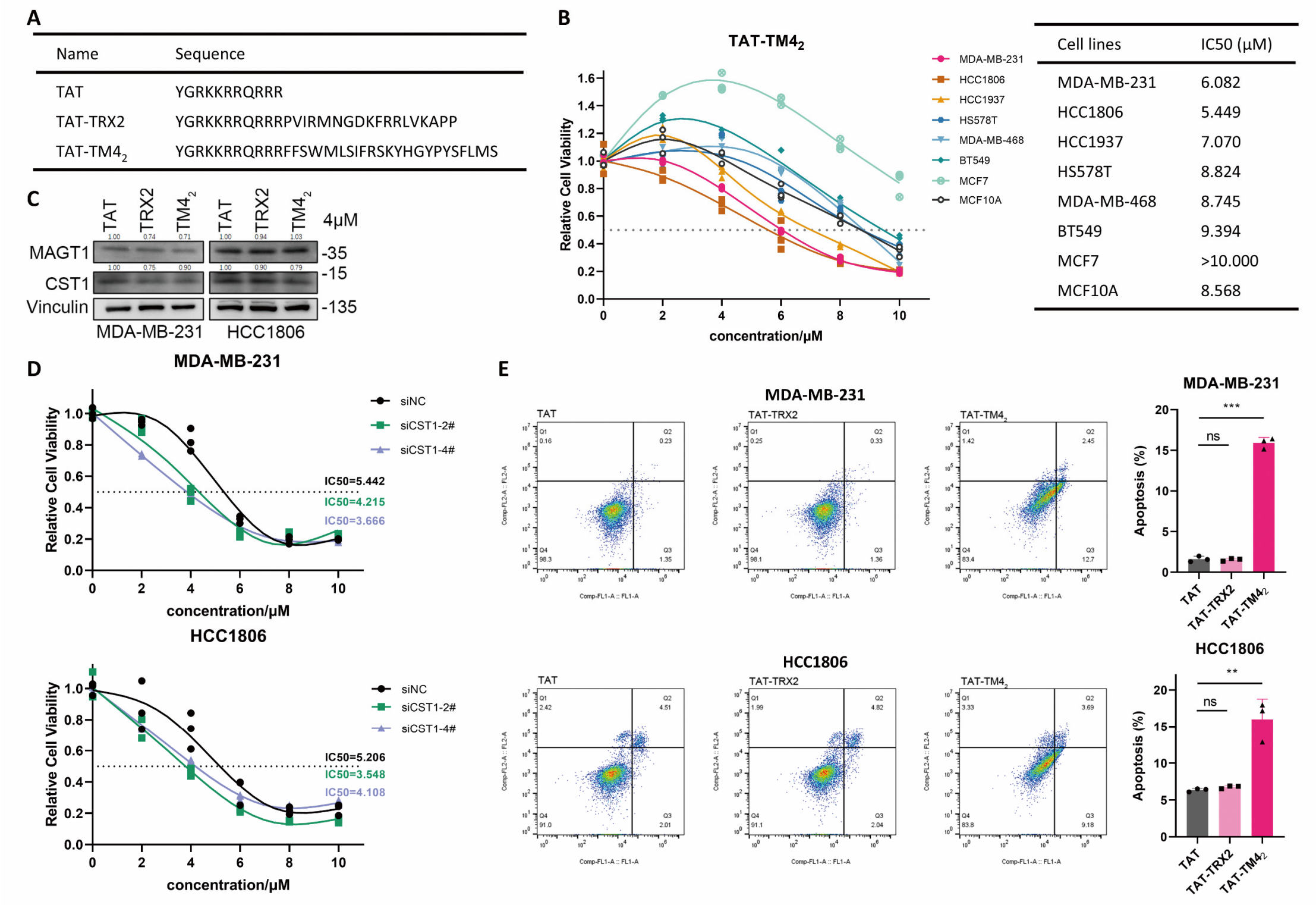

Fig. S7 CCP TAT-TM4$_2$ exhibited significant anti-tumor activity.

A Sequences of the three CCPs TAT, TAT-TRX2 and TAT-TM4$_2$.

B CCK8 assay analyzed the IC50 of TAT-TM4$_2$ in different breast cancer cell lines. Data are presented as the mean ± SD (n=3).

C Western blotting for analyzing the expression of CST1 and MAGT1 in breast cancer cell lines MDA-MB-231 and HCC1806 treated with different CCPs. Total Vinculin was used as a loading control.

D CCK8 assay analyzed the cell viability of CST1 knock-down MDA-MB-231 and HCC1806 cell lines after treatment with TAT-TM4$_2$. Data are presented as the mean ± SD (n=3).

E Cell apoptosis in MDA-MB-231 and HCC1806 cell lines treated with CCPs by flow cytometry. Data are presented as the mean ± SD (n=3).
